# Diacylglycerol lipase alpha promotes hepatocellular carcinoma progression and induces lenvatinib resistance by enhancing YAP activity

**DOI:** 10.1038/s41419-023-05919-5

**Published:** 2023-07-06

**Authors:** Yu-Chuan Yan, Guang-Xiao Meng, Chun-Cheng Yang, Ya-Fei Yang, Si-Yu Tan, Lun-Jie Yan, Zi-Niu Ding, Yun-Long Ma, Zhao-Ru Dong, Tao Li

**Affiliations:** 1 Department of General Surgery, Qilu Hospital of Shandong University, 250012 Jinan, China; 2 Laboratory of Basic Medical Sciences, Qilu Hospital of Shandong University, 250012 Jinan, China; 3 Key Laboratory for Experimental Teratology of the Ministry of Education, Key Laboratory of Infection and Immunity of Shandong Province, Department of Immunology, School of Basic Medical Sciences, Cheeloo Medical College of Shandong University, 250012 Jinan, China

**Keywords:** Liver cancer, Targeted therapies, Cancer therapeutic resistance

## Abstract

As an important hydrolytic enzyme that yields 2-AG and free fatty acids, diacylglycerol lipase alpha (DAGLA) is involved in exacerbating malignant phenotypes and cancer progression, but the role of the DAGLA/2-AG axis in HCC progression remains unclear. Here, we found that the upregulation of components of the DAGLA/2-AG axis in HCC samples is correlated with tumour stage and patient prognosis. In vitro and in vivo experiments demonstrated that the DAGLA/2-AG axis promoted HCC progression by regulating cell proliferation, invasion and metastasis. Mechanistically, the DAGLA/2AG axis significantly inhibited LATS1 and YAP phosphorylation, promoted YAP nuclear translocation and activity, and ultimately led to TEAD2 upregulation and increased PHLDA2 expression, which could be enhanced by DAGLA/2AG-induced activation of the PI3K/AKT pathway. More importantly, DAGLA induced resistance to lenvatinib therapy during HCC treatment. Our study demonstrates that inhibiting the DAGLA/2-AG axis could be a novel therapeutic strategy to inhibit HCC progression and enhance the therapeutic effects of TKIs, which warrant further clinical studies.

## Introduction

Hepatocellular carcinoma (HCC) is the most common subtype of primary liver cancer [1] and the third leading cause of cancer-related death worldwide [2]. Postoperative recurrence and metastasis lead to unsatisfactory prognosis in HCC patients who undergo radical surgery [3, 4]. In recent years, molecular targeted therapy and immunotherapy have greatly improved HCC treatment. However, no more than 20% of HCC patients benefit from these treatments because of intrinsic or acquired drug resistance [5, 6]. Elucidating critical events in the promotion of cancer development will enhance the understanding of the molecular mechanisms underlying HCC progression and thereby contribute to the development of novel targeted therapies.

The endocannabinoid system (ECS) is a complex intercellular communication system, and it is composed of bioactive lipid derivatives with binding affinity, called endocannabinoids (eCBs); a heterogeneous class of receptors; and a complex of enzymes that are responsible for the synthesis, transport, and hydrolysis of eCBs [7]. Dysregulation of the ECS can not only affect cancer cell proliferation, apoptosis, autophagy, invasion, metastasis, angiogenesis, chemoresistance and epithelial–mesenchymal transition (EMT) [8], but also modulate the functional behaviour of components of the tumour microenvironment including immune cells, endothelial cells and stromal components [9].

As an endogenous cannabinoid receptor agonist, 2-arachidonoylglycerol (2-AG) has been found to be involved in cancer pathophysiology and progression. For example, there are significantly increased 2-AG levels in late-stage diffuse large B-cell lymphomas, endometrial carcinoma and glioma tissues [10–13]. The role of 2-AG differs in different malignant tumours. Despite its high concentration in cancer cells, 2-AG exerts antiproliferative and anti-invasive effects on androgen-independent prostate cancer cells [14]. On the other hand, 2-AG could significantly enhance the immunosuppressive microenvironment by increasing the suppressive population of myeloid-derived suppressor cells to promote cancer progression [15]. In addition, 2-AG can be regulated by monoacylglycerol lipase (MAGL) to influence lung cancer cell invasion and metastasis [16].

As an important hydrolytic enzyme that yields 2-AG and free fatty acids, diacylglycerol lipase alpha (DAGLA) is required for axonal growth during brain development and for retrograde synaptic signalling at mature synapses [17]. DAGLA (-/-) animals showed an 80% reduction in 2-AG levels in the brain, and the resulting behavioural and physiological changes increased behavioural despair and reduced hippocampal neurogenesis [18]. DAGLA is also involved in exacerbating malignant phenotypes and contributing to oral squamous cell carcinoma progression by regulating the cell cycle [19]. To date, the role of the DAGLA/2-AG axis in HCC remains unclear.

Here, we reported that the DAGLA/2-AG axis was significantly higher in HCC tissues than in adjacent nontumor tissues and played an oncogenic role in promoting HCC cell proliferation, migration and invasion by inducing the EMT process and downregulating p57 expression. Mechanistically, the DAGLA/2-AG axis significantly inhibited LATS1 and YAP phosphorylation, promoted YAP nuclear translocation and activity, led to TEAD2 upregulation and increased PHLDA2 expression. YAP activity could be enhanced by DAGLA/2AG-induced activation of the PI3K/AKT pathway. More importantly, DAGLA could also induce lenvatinib resistance in HCC.

## Results

### Upregulation of the DAGLA/2-AG axis in HCC samples is correlated with advanced HCC tumour stage and poor patient prognosis

**Fig. 1:**
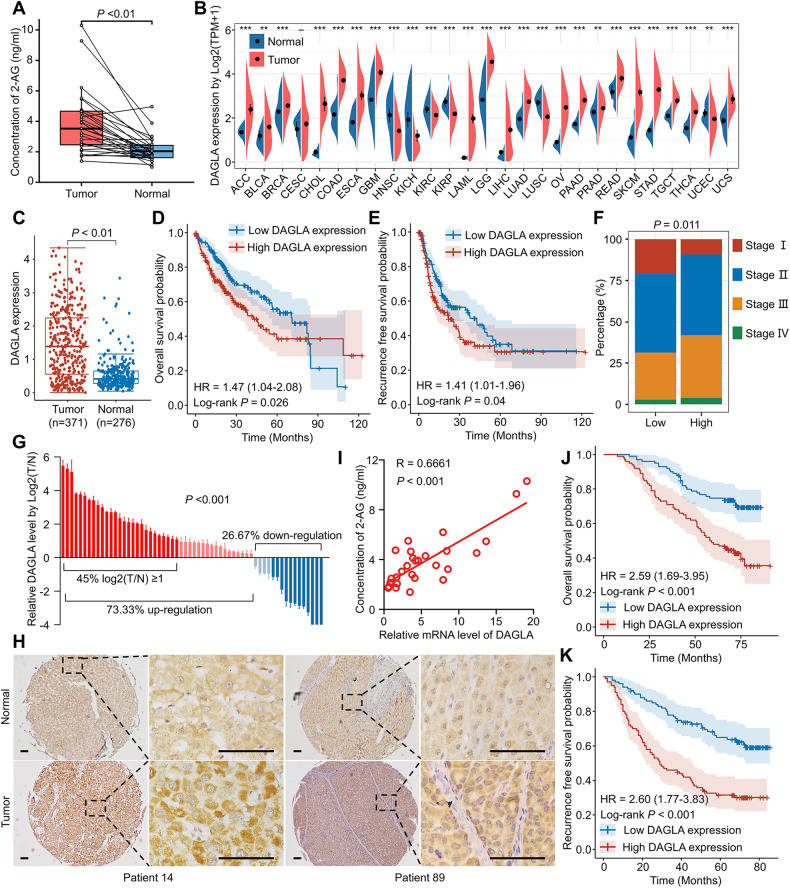
DAGLA is upregulated in HCC samples and is correlated with poor prognosis in HCC patients. **A** The difference in the 2-AG level between HCC tissues and adjacent nontumor tissues was detected by ELISA. **B** TCGA database showed that DAGLA mRNA was upregulated in a series of cancers, including HCC, compared with corresponding normal tissues. **C** TCGA and GTEx databases revealed that the DAGLA mRNA level was higher in tumour tissues than in normal liver tissues. **D**, **E** Kaplan–Meier analysis demonstrated that the DAGLA level was predictive of OS and RFS in HCC patients. **F** The proportion of stage III HCC patients in the high DAGLA group was markedly higher than that in the low DAGLA group. **G** qRT–PCR revealed that DAGLA was significantly upregulated in paired HCC tissue samples. **H** The change in DAGLA expression in representative HCC samples in a TMA by IHC staining. Scale bars, 100 μm. **I** DAGLA mRNA expression was positively correlated with the 2-AG concentration in HCC tissues. **J**, **K** Kaplan–Meier analysis of TMA patients indicated that high DAGLA expression in HCC tissues was correlated with worse OS and RFS.

We began our study by detecting the 2-AG difference in HCC and adjacent nontumor tissue samples. The 2-AG level in HCC was significantly higher than that in adjacent nontumor tissues (Fig. 1A). To determine why 2-AG was increased in HCC tissues, we focused on the key 2-AG biosynthesis enzyme DAGLA. The Cancer Genome Atlas (TCGA) and the Genotype–Tissue Expression (GTEx) database indicated that the DAGLA mRNA level was significantly increased compared with that in normal tissues in multiple malignancies, including HCC (Fig. 1B, C). The high DAGLA mRNA level in HCC tissues was positively correlated with shorter OS and RFS (Fig. 1D, E). The proportion of stage III patients in the high DAGLA group was significantly higher than that in the low DAGLA group (Fig. 1F). Our qRT–PCR and IHC staining results demonstrated that DAGLA mRNA and protein levels were greatly increased in HCC tissues compared with adjacent normal liver tissues (Fig. 1G, H). Moreover, the DAGLA mRNA level was positively correlated with the 2-AG level in HCC samples (Fig. 1I).

IHC staining revealed that DAGLA was expressed in both the cytoplasm and nucleus. Kaplan–Meier survival analysis indicated that patients with higher DAGLA levels in the cytoplasm had a worse prognosis than those with lower DAGLA levels (Fig. 1J, K). TMA clinical data revealed a statistically significant correlation between DAGLA levels and several clinicopathological features, such as sex, vascular invasion, tumour number and tumour differentiation (Supplementary Table S4, Supplementary Fig. S1A–D). Univariate and multivariate analyses identified DAGLA expression in HCC as an independent predictor of OS and RFS (Supplementary Tables S5 and S6). These results collectively suggest that DAGLA may function as an oncogene and a valuable prognostic predictor in HCC.

### The DAGLA/2-AG axis regulates HCC cell proliferation, migration and invasion in vitro and in vivo

**Fig. 2:**
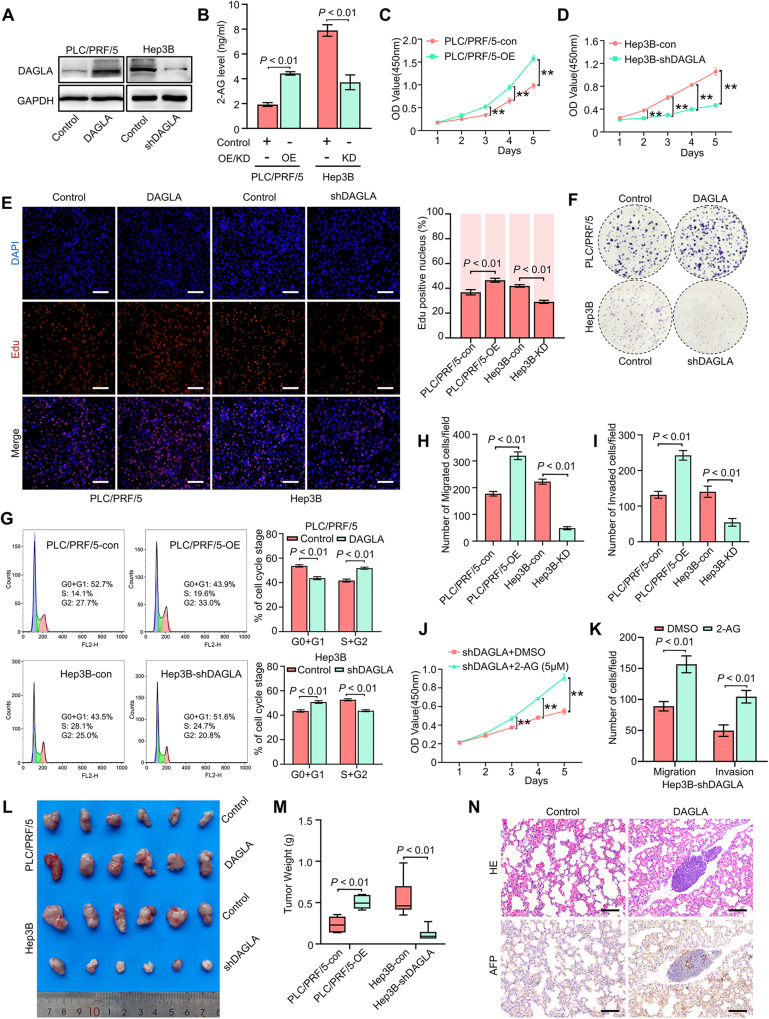
High DAGLA expression promotes HCC progression in vitro and in vivo. **A** Western blotting confirmed the OE and KD efficiencies of DAGLA. **B** The 2-AG level in PLC/PRF/5-OE and Hep3B-KD cells was determined by ELISA. **C**–**F** CCK-8, EdU incorporation and colony formation assays were used to determine the role of DAGLA in HCC cell proliferation and colony formation. **P < 0.01. Scale bars, 100 μm. **G** Flow cytometric analysis demonstrated that Hep3B-KD induced G1/S arrest in Hep3B cells and that PLC/PRF/5-OE increased the proportion of PLC/PRF/5 cells in the S/G2 phase. **H**, **I** Transwell assays showed that DAGLA promoted the migration and invasion of HCC cells. **J** CCK-8 assays were used to verify the effect of exogenous 2-AG (5 µM) on Hep3B-KD cells. **P < 0.01. **K** Transwell assays were used to evaluate the effect of exogenous 2-AG on the migration and invasion abilities of Hep3B-KD cells. **L**, **M** Xenograft tumours derived from PLC/PRF/5-OE PLC/PRF/5 cells were significantly larger and tumours derived from Hep3B-KD Hep3B cells were significantly smaller than those derived from the corresponding control cells. **N** DAGLA promoted the lung metastasis of HCC cells in vivo. Scale bars, 100 μm.

To investigate the role of the DAGLA/2-AG axis in HCC progression, qRT–PCR and Western blotting were used to evaluate the mRNA and protein levels of DAGLA in human HCC cell lines, respectively (Supplementary Fig. S2A, B). Then, lentivirus-mediated transduction was used to construct DAGLA-overexpressing PLC/PRF/5 (PLC/PRF/5-OE) and DAGLA-knockdown Hep3B (Hep3B-KD) cell lines (Fig. 2A, Supplementary Fig. S2C, D). We found that the 2-AG level in Hep3B-KD cells was significantly lower than that in Hep3B-Con cells, while PLC/PRF/5-OE cells had a significantly higher 2-AG level than control cells (Fig. 2B). The proliferation and clonogenic abilities of Hep3B cells were impaired when DAGLA expression was substantially inhibited, and these abilities were enhanced when DAGLA was overexpressed in PLC/PRF/5 cells (Fig. 2C–F, Supplementary Fig. S2E). These results were confirmed by IF staining for Ki-67 (Supplementary Fig. S2F). Flow cytometric analysis showed that DAGLA downregulation in Hep3B cells induced G1/S arrest, with an increasing proportion of cells in the G1 phase (Fig. 2G). In addition, downregulation of DAGLA reduced the migration and invasion abilities of HCC cells, while DAGLA overexpression could remarkably enhance HCC cells’ migration and invasion abilities (Fig. 2H, I, Supplementary Fig. S2G, H). To determine the critical role of 2-AG in DAGLA biological function, exogenous 2-AG was added to Hep3B-KD cells. The CCK-8 and Transwell assays demonstrated that the addition of exogenous 2-AG could reverse the DAGLA-KD-induced inhibition of cell proliferation, invasion and metastasis in Hep3B cells (Fig. 2J, K).

Then, Hep3B-Con, Hep3B-KD, PLC/PRF/5-Con, and PLC/PRF/5-OE cells were injected into nude mice. Five weeks after transplantation, the xenograft tumours derived from Hep3B-KD cells were significantly smaller than those derived from control cells, while the tumours in the PLC/PRF/5-OE group were larger than those in the PLC/PRF/5-Con group (Fig. 2L, M, Supplementary Fig. S2I). In addition, DAGLA significantly promoted the lung metastasis of HCC cells (Fig. 2N). Furthermore, the results of IHC staining for Ki-67 were similar to those of the in vitro IF assays (Supplementary Fig. S2J). Therefore, we speculate that the DAGLA/2-AG axis functions as an important tumour activator to promote HCC cell proliferation, invasion and metastasis in vivo and in vitro.

### The oncogenic role of the DAGLA/2-AG axis in HCC mainly depends on the Hippo and PI3K/AKT signalling pathways

**Fig. 3:**
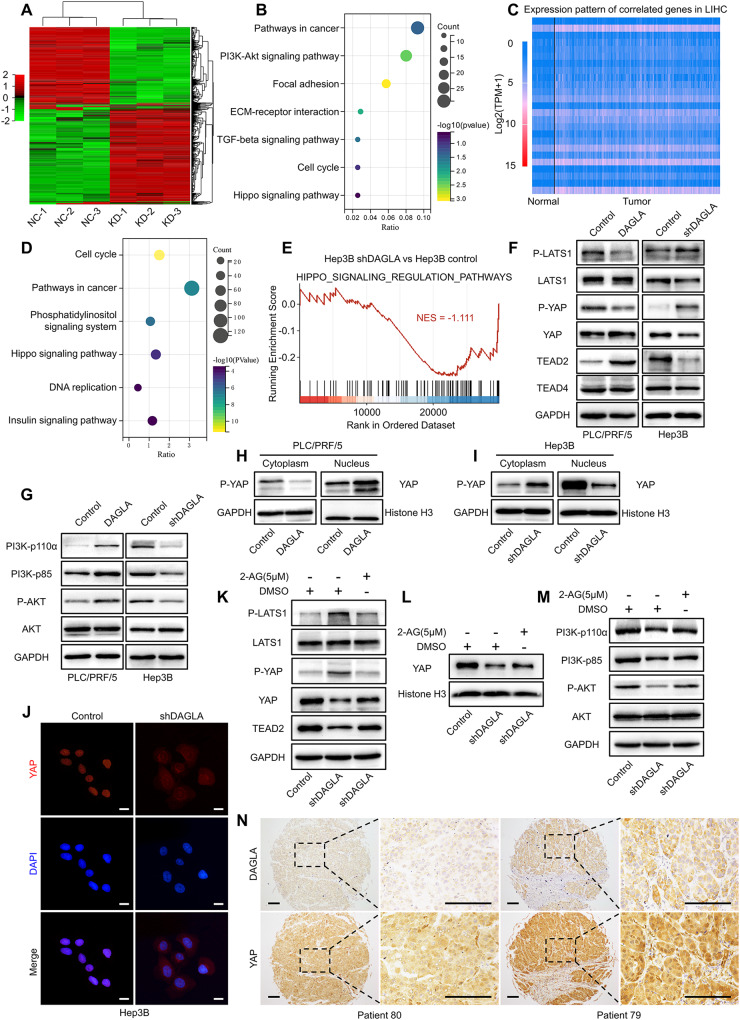
The tumorigenic role of DAGLA in HCC is mediated by the Hippo and PI3K/AKT signalling pathways. **A** Heatmap of the DEGs in Hep3B-shDAGLA cells compared with control cells. **B** KEGG analysis of downregulated DEGs in Hep3B-shDAGLA cells revealed the potential signalling pathways regulated by DAGLA in HCC. **C** Heatmap of the representative genes whose expression was correlated with DAGLA expression identified by TCGA database analysis with the filtering criteria of a correlation coefficient ≥0.3 and a *P*-value < 0.05. **D** KEGG pathway enrichment analysis of the gene set positively correlated with DAGLA showed the enriched signalling pathways correlated with DAGLA in HCC. **E** Gene set enrichment analysis of Hep3B-KD and control cells. The normalised enrichment score (NES) is shown. **F**, **G** Western blotting analysis confirmed the effects of DAGLA OE and KD on the Hippo and PI3K/AKT signalling pathways in HCC. **H**, **I** A nuclear/cytoplasmic protein extraction assay revealed that Hep3B-shDAGLA and PLC/PRF/5-DAGLA increased and decreased the phosphorylation level of YAP in the cytoplasm, respectively. **J** The IF assay indicated that DAGLA drove the nuclear translocation of YAP in HCC cells. Scale bars, 10 μm. **K** Hep3B-shDAGLA cells were treated with exogenous 2-AG (5 µM) for 48 h, and WB analysis was performed to detect changes in the expression of the indicated proteins. **L** WB showed that exogenous 2-AG rescued the low nuclear YAP level resulting from DAGLA knockdown. **M** WB was performed to detect the expression of the indicated proteins. **N** IHC staining of the HCC TMA revealed the correlation between high DAGLA expression and YAP nuclear translocation. Scale bars, 150 μm.

To elucidate the molecular mechanisms of the DAGLA/2-AG axis in HCC progression, RNA sequencing was used to identify differentially expressed genes (DEGs) between HCC cells with different levels of DAGLA. Our results revealed that 1619 DEGs (fold change ≥ 2 and *P* < 0.05), including 900 upregulated and 719 downregulated DEGs, were identified between the Hep3B-con and Hep3B-KD cells (Fig. 3A, Supplementary Fig. S3A). Kyoto Encyclopedia of Genes and Genomes (KEGG) and Gene Ontology (GO) analyses showed that downregulated DEGs were enriched in signalling pathways such as the PI3K/Akt signalling pathway, Hippo signalling pathway, focal adhesion, positive regulation of cell proliferation, cell migration and EMT (Fig. 3B, Supplementary Fig. S3B). The KEGG analysis of upregulated DEGs and the remaining GO analysis results of downregulated DEGs are provided in the supplementary figures (Supplementary Fig. S3C–E).

Then, we analysed the genes significantly correlated with DAGLA mRNA levels in HCC samples from the TCGA database. A total of 4475 positively correlated genes and 49 negatively correlated genes were identified (Fig. 3C). KEGG analysis showed that the positively correlated genes were enriched in signalling pathways such as the Hippo signalling pathway and insulin signalling pathway, as well as several biological signalling systems such as the cell cycle, DNA replication and phosphatidylinositol signalling (Fig. 3D). Gene set enrichment analysis (GSEA) using curated gene sets from RNA-seq indicated significant enrichment for a gene set encoding Hippo signalling regulation pathways (Fig. 3E). The KEGG pathway enrichment analysis results for the negatively correlated genes are presented in supplementary figures (Supplementary Fig. S3F).

As a master regulator of tumorigenesis, progression and therapeutic resistance, the Hippo pathway is notable in many cancers and relays signals produced by extracellular and intracellular events to regulate cell behaviour and functions [20, 21]. Positive feedback was observed between the Hippo and PI3K/AKT pathways in nonalcoholic fatty liver disease [22]. Based on the results of RNA-seq and correlation analysis of DAGLA expression in HCC, we hypothesised that the DAGLA/2-AG axis might play an oncogenic role by cross-regulating the Hippo and PI3K/AKT pathways.

To verify this hypothesis, we examined the changes in key components in the Hippo and PI3K/AKT pathways. Hep3B-KD induced phosphorylation of LATS1 and YAP as well as downregulation of TEAD2, while PLC/PRF/5-OE inhibited LATS1 and YAP phosphorylation, leading to YAP nuclear translocation and TEAD2 upregulation, which was accompanied by activation of the PI3K/AKT pathway (Figs. 3F–J, S3G–J). Exogenous 2-AG not only inhibited LATS1 phosphorylation and then promoted YAP nuclear translocation (Fig. 3K, L, Supplementary Fig. S3K, L) but also led to the reactivation of PI3K/AKT signalling in Hep3B-KD cells (Fig. 3M, Supplementary Fig. S3M). In addition, xenograft Hep3B-KD tumours exhibited lower YAP expression, while PLC/PRF/5-OE tumours had greater YAP nuclear translocation. (Supplementary Fig. S3N). In addition, HCC patients with high DAGLA expression exhibited a higher incidence of YAP nuclear translocation events and a worse prognosis (Fig. 3N).

### YAP-mediated crosstalk between the PI3K/AKT and Hippo signalling pathways plays a crucial role in DAGLA/2-AG axis-induced HCC progression

**Fig. 4:**
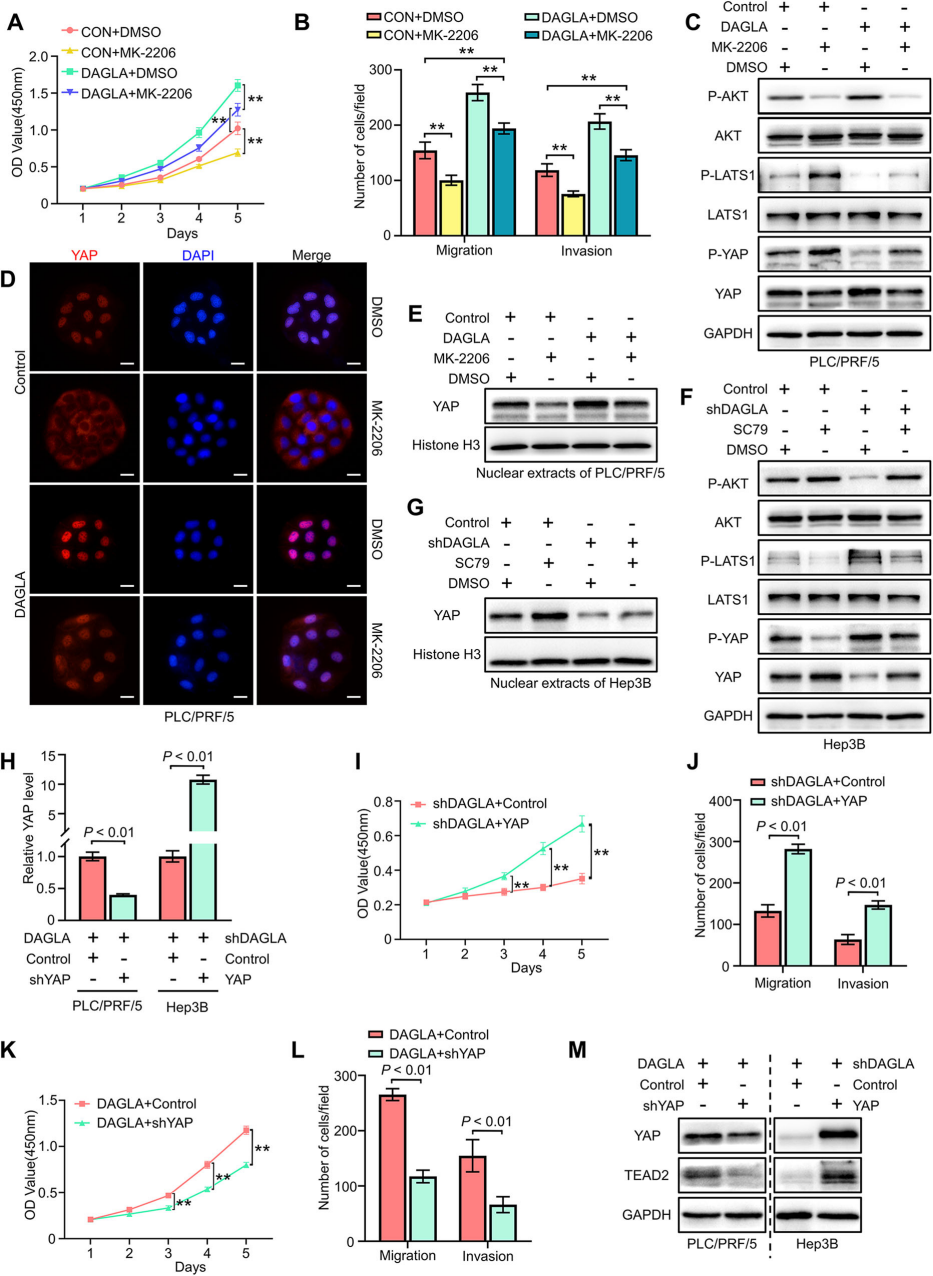
DAGLA facilitates HCC progression by regulating the Hippo and PI3K/AKT pathways. **A**, **B** CCK-8 and Transwell assays were used to determine the effect of MK-2206 (10 μM, DMSO as a control) on PLC/PRF/5-DAGLA cells. **P < 0.01, ***P < 0.001. **C** Western blotting analysis revealed that MK-2206 treatment (48 h) increased the p-LATS1 and p-YAP levels in PLC/PRF/5-DAGLA cells to some extent. **D** IF staining and Western blotting analysis (**E**) showed that MK-2206 slightly blocked YAP nuclear translocation in PLC/PRF/5-DAGLA cells. Scale bars, 10 μm. **F** Western blotting analysis was used to determine the effect of SC79 (10 μM, 1 h; DMSO as the control) on the levels of the indicated proteins in Hep3B-shDAGLA cells. **G** WB demonstrated that SC79 facilitated YAP nuclear transport, which was partially inhibited by DAGLA downregulation. **H** qRT–PCR confirmed the efficiency of cotransfection of YAP and DAGLA modulation constructs. **I**–**L** CCK-8 and Transwell assays were used to explore the effects of YAP OE or KD on Hep3B-shDAGLA or PLC/PRF/5-DAGLA cells, respectively. **P < 0.01. **M** WB indicated the regulation of the Hippo signalling pathway in cell lines cotransfected with YAP and DAGLA modulation constructs.

To determine the relationship between the PI3K/AKT and Hippo pathways, PLC/PRF/5-OE HCC cells were treated with the AKT inhibitor MK-2206 (10 μM). After 48 h of treatment, MK-2206 obviously suppressed the proliferation and invasion of HCC cells (Fig. 4A, B). In addition, MK-2206 partially inhibited DAGLA-induced activation and nuclear translocation of YAP in PLC/PRF/5 cells (Fig. 4C–E, Supplementary Fig. S4A, B). Then, Hep3B-KD cells were treated with the AKT agonist SC79 (10 μM) for 1 h, and we found that SC79 notably delayed Hep3B-KD-induced p-YAP accumulation and facilitated YAP nuclear translocation to a limited extent (Fig. 4F, G, Supplementary Fig. S4C, D).

Considering the crucial role of YAP signalling in DAGLA-induced HCC progression [23], we overexpressed YAP in Hep3B-KD cells and knocked down YAP in PLC/PRF/5-OE cells (Fig. 4H). We found that YAP OE or KD could reverse the DAGLA down/upregulation-induced proliferation and invasion inhibition or promotion in HCC cells, respectively (Fig. 4I–L). Western blotting analysis showed that YAP OE and KD induced the activation or inhibition of TEAD2, respectively (Fig. 4M, Supplementary Fig. S4E).

### The DAGLA/2-AG axis enhances HCC progression through YAP/TEAD2-induced PHLDA2 transcriptional activation and expression

**Fig. 5:**
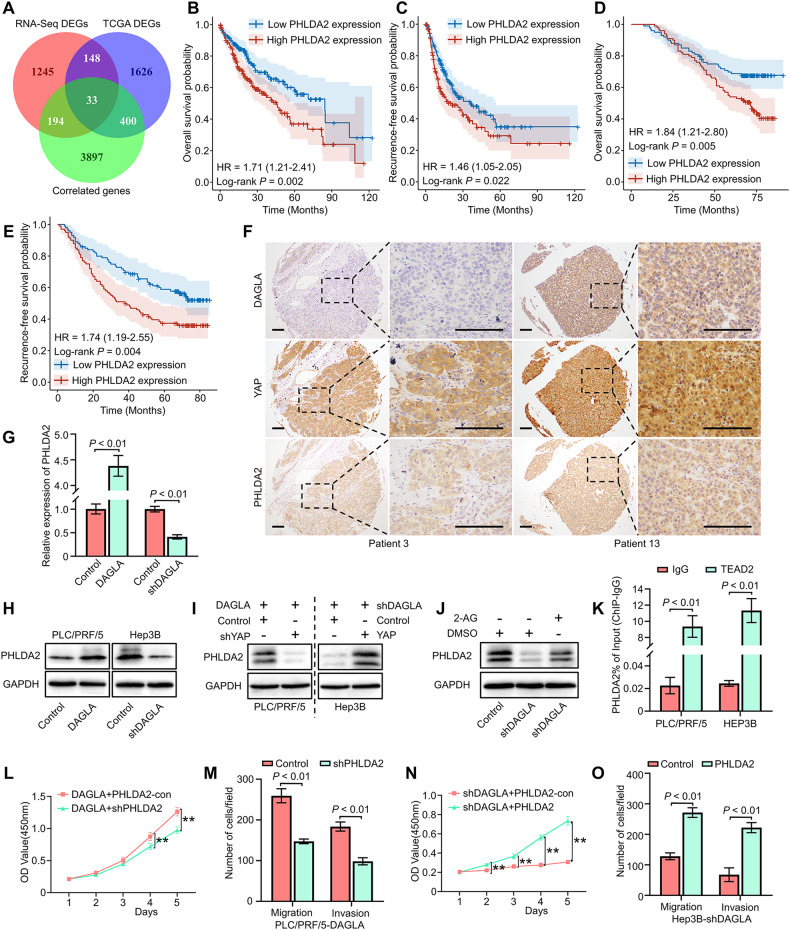
DAGLA promotes HCC progression by upregulating PHLDA2 expression induced by YAP nuclear translocation. **A** The intersection of DEGs identified by TCGA database analysis, RNA-seq and DAGLA-correlated genes is shown in a Venn diagram. **B**, **C** TCGA database analysis indicated that patients with high PHLDA2 expression had worse OS and RFS. **D**, **E** The results of TMA IHC staining revealed that high PHLDA2 expression was associated with worse OS and RFS in HCC patients. **F** IHC staining of the HCC TMA demonstrated that high DAGLA levels were associated not only with higher YAP nuclear translocation but also with high PHLDA2 expression. Scale bars, 150 μm. **G**, **H** qRT–PCR and WB confirmed that DAGLA could regulate PHLDA2 expression in HCC cells. **I** WB showed the expression of PHLDA2 in Hep3B-shDAGLA+YAP-OE cells and PLC/PRF/5-DAGLA+YAP-KD cells. **J** WB showed that exogenous 2-AG rescued the low PHLDA2 expression induced by DAGLA knockdown. **K** ChIP‒qPCR revealed that TEAD2 bound to the PHLDA2 promoter in HCC cells. **L**–**O** CCK-8 and Transwell assays verified the biological effect of PHLDA2 in Hep3B-KD/OE cells. **P < 0.01.

To further elucidate the molecular mechanism by which the DAGLA/2-AG axis participates in HCC progression, we comprehensively analysed the overlap among the 1619 DEGs (fold change ≥2, *P* < 0.05) of RNA-seq, 2207 DEGs (fold change ≥ 2, *P* < 0.05) between HCC and nontumor tissues and 4524 DAGLA-correlated genes (R ≥ 0.3 and *P* < 0.05) from the TCGA database. A total of 33 genes, including PHLDA2, were identified as potential downstream molecules of the DAGLA/2-AG axis (Fig. 5A, Supplementary Table S7).

PHLDA2 is an imprinted gene associated with several malignancies and can promote colorectal cancer proliferation and metastasis [24]. The TCGA database and IHC staining showed that PHLDA2 expression was upregulated in HCC tissues, and the PHLDA2 mRNA level in HCC samples was correlated with HCC patients’ prognosis and the DAGLA level (Fig. 5B, C, Supplementary Fig. S5A–C). IHC staining of the HCC TMA confirmed that the protein level of PHLDA2 was not only correlated with HCC patients’ prognosis but also positively related to the expression of DAGLA and YAP in HCC samples (Fig. 5D–F).

To determine whether the DAGLA/2-AG axis enhances HCC progression by upregulating PHLDA2 expression, we examined the changes in PHLDA2 expression in HCC cells with different DAGLA levels. Our results confirmed that PHLDA2 expression could be regulated by DAGLA in vitro and in vivo (Fig. 5G, H, Supplementary Fig. S5D, E). YAP reversed the DAGLA-KD-induced decrease in PHLDA2 expression in Hep3B cells, while YAP-KD inhibited the DAGLA-KD-induced activation of PHLDA2 in PLC/PRF/5 cells (Fig. 5I, Supplementary Fig. S5F, G). In addition, exogenous 2-AG upregulated PHLDA2 expression in Hep3B-KD cells (Fig. 5J, Supplementary Fig. S5H).

Inactivation of Hippo signalling leads to the dephosphorylation and nuclear translocation of YAP/TAZ, which then bind to TEAD family proteins and ultimately activate the transcription of downstream target genes. Among the TEAD1-4 genes, the TEAD2 mRNA level was positively correlated with the DAGLA and PHLDA2 mRNA levels, as well as with poor OS and RFS in HCC patients (Supplementary Fig. S5I–L). DAGLA regulated TEAD2 transcription and expression in HCC cells in vitro and in vivo (Supplementary Fig. S5M). The JASPAR network (https://jaspar.genereg.net/) and ChIP‒qPCR demonstrated the binding of the PHLDA2 transcriptional promoter region with TEAD2 in HCC cells (Fig. 5K).

To determine the role of PHLDA2 in the DAGLA/2-AG axis function, PHLDA2 knockdown (PHLDA2-KD) and overexpression (PHLDA2-OE) cell lines were constructed in PLC/PRF/5 and Hep3B cells, respectively (Supplementary Fig. S5N). PHLDA2 downregulation impaired the proliferation and invasion abilities of HCC cells, while PHLDA2 overexpression could strengthen HCC cells’ proliferation and invasion abilities in vitro (Supplementary Fig. S5O–R). Subsequently, PHLDA2-OE and PHLDA2-KD lentiviruses were used to infect Hep3B-DAGLA-KD and PLC/PRF/5-DAGLA-OE cells, respectively. The corresponding assays revealed that PHLDA2-OE rescued the defective proliferation and metastasis potential in Hep3B-DAGLA-KD cells while inhibiting PHLDA2 blocked the DAGLA-OE-induced increase in the malignant phenotype in vitro (Fig. 5L–O). These results demonstrated that the DAGLA/2-AG axis could promote HCC progression through YAP/TEAD2-induced PHLDA2 transcriptional activation and expression.

### DAGLA/2-AG axis promotes HCC progression by inducing the EMT process and downregulating p57 expression

**Fig. 6:**
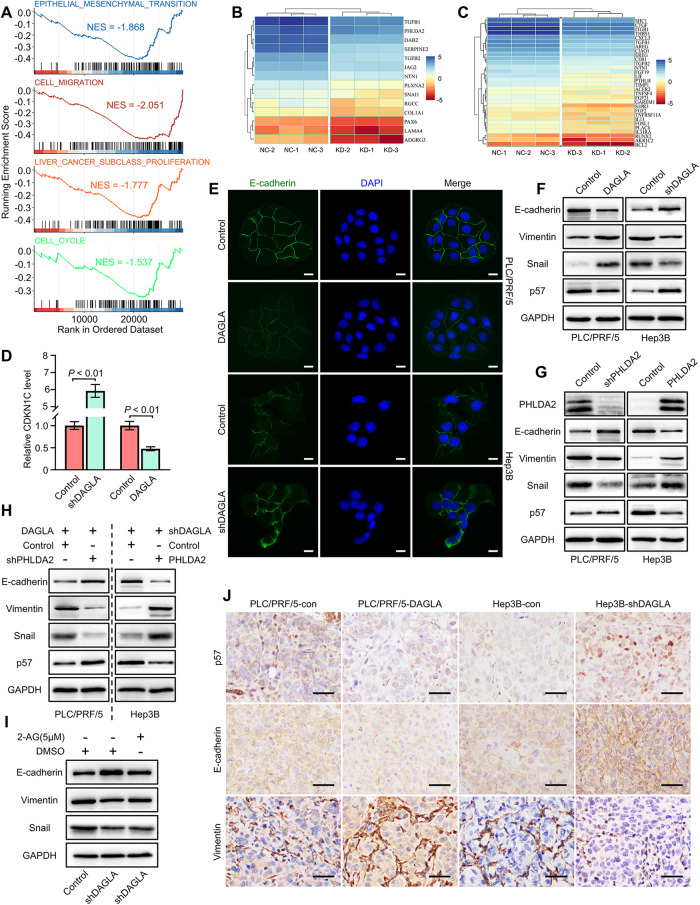
PHLDA2 promotes HCC progression by facilitating cell proliferation and inducing the EMT process. **A** GSEA results of gene sets related to EMT, cell migration, liver cancer proliferation and cell cycle related in Hep3B-shDAGLA cells compared with the control group. **B** Heatmap of the expression of EMT- and cell migration-related genes in Hep3B-shDAGLA cells compared with the control group. **C** Heatmap of the expression of liver cancer proliferation- and cell cycle-related genes in Hep3B-shDAGLA cells compared with the control group. **D** The CDKN1C (p57) mRNA level was determined by qRT–PCR. **E** IF staining revealed the change in the E-cadherin levels in DAGLA-OE/KD cells. Scale bars, 10 μm. **F** Western blotting revealed the influence of DAGLA on the protein levels of key EMT mediators and p57. **G** Western blotting analysis clarified the effect of PHLDA2 on the expression of key EMT mediators and p57. **H** The levels of p57 and EMT-related proteins were verified by WB in PLC/PRF/5-DAGLA+PHLDA2-KD cells and Hep3B-shDAGLA+PHLDA2-OE cells. **I** Western blotting analysis showed the influence of exogenous 2-AG on EMT mediator expression in Hep3B-shDAGLA cells. **J** IHC staining demonstrated variations in the levels of p57, E-cadherin and vimentin in mouse xenograft tumours. Scale bars, 50 μm.

To elucidate the underlying mechanism of the DAGLA/2-AG axis in HCC progression, GSEA was performed to verify the GO analysis results of RNA-seq data and revealed that gene sets encoding epithelial–mesenchymal transition, cell migration, liver cancer proliferation and the cell cycle were notably downregulated in Hep3B-DAGLA-KD cells (Fig. 6A–C). Subsequently, we confirmed that critical mediators of the EMT process and p57 expression were regulated by DAGLA and PHLDA2 in HCC cells (Fig. 6D–G, Supplementary Fig. S6A, B). As a downstream target of the DAGLA-activated YAP/TEAD2 axis, PHLDA2 could induce the EMT process and inhibit p57 activity in HCC cells (Fig. 6H, Supplementary Fig. S6C). Exogenous 2-AG led to the downregulation of E-cadherin expression as well as the upregulation of snail and vimentin expression in Hep3B-DAGLA-KD cells (Fig. 6I, Supplementary Fig. S6D). IHC staining demonstrated low p57 or E-cadherin and high vimentin levels in xenograft tumours derived from PLC/PRF/5-DAGLA cells but high p57 or E-cadherin and low vimentin levels in xenograft tumours from Hep3B-DAGLA-KD cells compared with the control tumours (Fig. 6J).

### DAGLA modulated HCC therapeutic sensitivity to tyrosine kinase inhibitors

Currently, tyrosine kinase inhibitors such as sorafenib and lenvatinib remain a crucial targeted therapy that is recommended for advanced HCC patients. However, the clinical efficacy of multikinase inhibitors has been severely weakened due to acquired resistance [3]. Previous studies indicated that HCC cells with an EMT phenotype may develop resistance to treatment with multikinase inhibitors [25]. Moreover, YAP signalling has been shown to modulate sensitivity to lenvatinib treatment [26, 27]. Our study elucidated the important role of DAGLA in HCC progression via 2-AG-induced YAP signalling and EMT, which prompted us to hypothesise that DAGLA may regulate the sensitivity of HCC cells to multikinase inhibitor treatment.

**Fig. 7:**
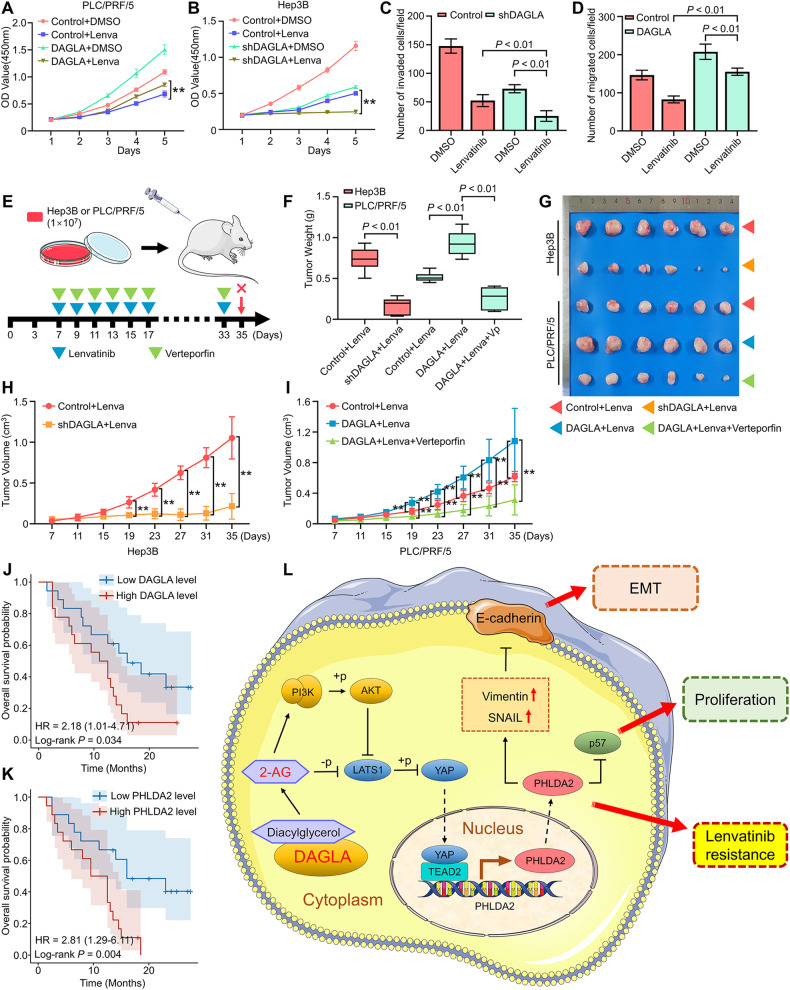
DAGLA could modulate sensitivity to multikinase inhibitor therapy. **A**–**D** CCK-8 and Transwell assays showed the effect of DAGLA OE and KD on the proliferation and invasion ability of HCC cells treated with lenvatinib (10 μM). **E** Experimental scheme. **F**–**I** Xenograft tumours derived from DAGLA-knockdown cells and treated with lenvatinib (10 mg/kg) showed better tumour growth inhibition, while tumours derived from DAGLA-overexpressing cells showed significant lenvatinib resistance, which could be reversed by verteporfin (40 mg/kg). **J**, **K** Comparison of OS curves between 36 advanced recurrent HCC patients with high or low DAGLA and PHLDA2 levels treated with lenvatinib and TACE who underwent liver resection before combined therapy. **L** Schematic depiction of the mechanisms underlying the DAGLA/2-AG axis regulating HCC progression and lenvatinib resistance by enhancing YAP nuclear translocation and activity.

To confirm this hypothesis, CCK-8 and Transwell assays were performed, and the results confirmed that DAGLA overexpression reversed the Lenvatinib-induced inhibition of proliferation and invasion in PLC/PRF/5 cells, while DAGLA knockdown enhanced the sensitivity of Hep3B cells to lenvatinib treatment (Fig. 7A–D). Subsequently, subcutaneous tumour models were established in nude mice by using Hep3B and PLC/PRF/5 cells with different DAGLA levels in order to explore the effect of DAGLA expression and verteporfin (a YAP antagonist) on lenvatinib resistance. Drug treatments were administered by intraperitoneal injection as indicated (Fig. 7E). Compared with the control groups, the nude mice bearing DAGLA-knockdown tumours and receiving lenvatinib treatment showed better tumour growth inhibition, while nude mice bearing DAGLA-overexpressing tumours exhibited significant lenvatinib resistance, which could be strongly reversed by the combined treatment of verteporfin and lenvatinib (Fig. 7F–I).

The expression of DAGLA and PHLDA2 was observed in HCC tissues from 36 patients with advanced HCC recurrence who underwent partial liver resection and TACE treatment before lenvatinib treatment. Kaplan‒Meier analysis revealed that patients with high DAGLA and PHLDA2 levels had significantly shorter OS than those in the low DAGLA and PHLDA2 groups (Fig. 7J, K). Therefore, we confirmed that DAGLA together with PHLDA2 could modulate the therapeutic sensitivity of HCC to lenvatinib treatment.

## Discussion

eCBs, including 2-AG, anandamide and other bioactive lipid derivatives, are involved in several aspects of cancer pathophysiology, such as proliferation, metastasis, peritumoral inflammation and the immune microenvironment [28]. Previous studies have demonstrated that eCB profiles are dysregulated during cancer progression, particularly in metastatic tumours [29]. However, the underlying mechanism remains unclear. Our study proposed a molecular mechanism by which the ECS regulates tumour progression and metastasis. We found that the DAGLA/2-AG axis facilitates HCC cell proliferation and the EMT process by enhancing YAP nuclear translocation and activity. First, DAGLA expression was shown to be significantly upregulated in HCC tissues. The DAGLA/2-AG axis promoted HCC proliferation, migration and invasion in vitro and in vivo. Mechanistic analysis showed that the DAGLA/2-AG axis played an oncogenic role by regulating the nuclear translocation and activity of YAP, which could then bind to TEAD2 and increase the transcriptional activation of PHLDA2. More importantly, DAGLA was identified as a prognosis-associated target and was found to promote HCC progression by inducing the EMT process and suppressing p57 accumulation in vivo and in vivo (Fig. 7L). Clinically, the DAGLA level in HCC tissues was positively correlated with the incidence of YAP nuclear translocation and PHLDA2 expression. In addition, DAGLA could induce HCC resistance to lenvatinib. These results reveal that DAGLA functions as an oncogene in HCC progression and could be considered a potential therapeutic target in HCC.

The Hippo pathway is evolutionarily conserved and plays tumour-suppressive roles in various cancers arising from gastric, thoracic, gynaecological, genitourinary, skin, bone and brain tissues. YAP is the core component of the Hippo pathway, the activity of which is mainly regulated by post-translational modifications (PTM), in particular by phosphorylation on critical serine residues by LATS1/2. This PTM process directly inhibits the levels and activities of YAP via phosphorylation-mediated 14-3-3 binding and cytoplasmic retention [21, 23]. In the present study, we evaluated the effect of the DAGLA/2-AG axis on YAP and confirmed that nonphosphorylated YAP (the active form) was significantly enriched in the nuclear region. Interestingly, this effect not only appeared to regulate YAP nuclear translocation but also affected the overall expression level of YAP. Previous studies have shown that activation of the Hippo pathway inhibits YAP not only by retaining YAP in the cytoplasm but also by stimulating YAP degradation [30]. Moreover, the activation of the PI3K/AKT pathway could further promote YAP stability and nuclear localisation by SKP2-induced K63-linked polyubiquitination in a Hippo-independent manner [31, 32].

In addition to the NDR kinase family, which includes LATS1 and LATS2, several other signalling factors, such as AKT, Wnt, TGFβ, MAPK, GPCRs and metabolic networks, can also regulate YAP/TAZ activities in different contexts [33]. In HCC, hypoxia-induced Akt/Rac1-mediated fascin-1 upregulation could enhance the malignant properties of HCC by mediating YAP activation [34]. Moreover, YAP could strongly mediate the acetylation of Skp2 via AKT signalling, resulting in diploid–polyploid conversion and polyploid cell growth in HCC [35]. Our study provided evidence that the ECS is involved in the regulatory networks connecting the Hippo and PI3K/AKT signalling pathways; we identified the promoting effect of DAGLA on YAP nuclear localisation induced by regulating LATS1 and the PI3K/AKT signalling pathway.

EMT is an evolutionarily conserved developmental programme that confers metastatic properties on cancer cells, which then tend to detach from the primary tumour, acquire a mesenchymal morphology and invade through the extracellular stroma to form distant metastasis after recognising certain signals from intracellular and extracellular sources. Tumour cells undergoing EMT acquire stem cell properties, leading to the generation of cancer stem cells and marked therapeutic resistance [25, 36]. Elucidating the molecular mechanism by which EMT regulates HCC progression is crucial for inhibiting metastasis and improving the prognosis of HCC patients. Previous studies have revealed the regulation of the EMT process by Hippo/YAP signalling; YAP/TAZ can interact with snail and twist to enhance the invasive capabilities of cancer cells [37, 38]. Moreover, the EMT-inducing transcriptional repressor ZEB1 can function as a transcriptional coactivator of YAP, integrating Hippo signalling and EMT processes with similar cancer-promoting effects [39, 40].

PHLDA2 is a maternally imprinted gene associated with tumour progression and EMT in several malignancies, such as colorectal cancer and pancreatic ductal adenocarcinoma [24, 41]. However, the role and upstream regulatory mechanism of PHLDA2 in HCC remain unclear. Herein, we describe PHLDA2 as a prognosis-associated oncogene that can facilitate malignant behaviours such as proliferation and invasion in HCC. Mechanistically, we confirmed that PHLDA2 functions as the downstream effector of the YAP-TEAD2 transcriptional complex and verified its enhancement of EMT progression, including upregulation of snail and vimentin and suppression of E-cadherin expression, further supplementing the mechanistic networks of Hippo/YAP signalling in regulating the EMT process. In conclusion, we identified the DAGLA/2-AG axis as a crucial driver of tumour proliferation and the EMT process, resulting in the rapid progression and multikinase inhibitor resistance of HCC.

## Materials and methods

### Cells and cell culture

Huh7, MHCC97H, PLC/PRF/5 and Hep3B cells were gifts from Zhongshan Hospital, Fudan University, and STR genotyping was performed. Huh7, MHCC97H, and PLC/PRF/5 cells were maintained in high-glucose DMEM supplemented with 10% foetal bovine serum, penicillin (100 IU/ml) and streptomycin (100 μg/ml). Hep3B cells were maintained in MEM-alpha supplemented with 10% foetal bovine serum, penicillin (100 IU/ml) and streptomycin (100 μg/ml). All cells were cultured in 25-cm^2^ polystyrene culture flasks in a humidified atmosphere of 5% CO_2_ at 37 °C.

### Clinical specimens, patient follow-up, tissue microarray (TMA) and xenograft mouse models

These experiments were approved by the Ethics Committee of Qilu Hospital of Shandong University and were performed in accordance with the approved guidelines. The sample size is estimated on the basis of our previous publication [4]. Fresh human HCC and adjacent nontumor liver tissue samples were randomly collected from consecutive HCC patients undergoing curative resection at Qilu Hospital, Shandong University (Jinan, China). Before the operation, we obtained the patients’ informed consent for harvesting residual resected tissues after pathological diagnosis. The TMA was constructed using specimens from 200 HCC patients. Clinicopathological information was collected, and follow-up procedures were performed as described previously [4].

Male athymic BALB/c nude mice (5 weeks old, GemPharmatech, China) were raised under specific pathogen-free conditions and randomly divided into groups before injection. The growth of tumours that were derived from HCC cells was monitored following subcutaneous injection of cells into nude mice (1 × 10^7^ cells/mouse, 6 mice/group). Five weeks after injection, mice were sacrificed under anaesthesia, and the xenograft tumours were excised for further analysis. All the animal experiments were performed in accordance with the *Guide for the Care and Use of Laboratory Animals* established by the National Institutes of Health.

### RNA isolation and quantitative reverse transcription–PCR (qRT–PCR)

Total RNA was extracted from tissues and HCC cell lines by using TRIzol reagent (15596026, Invitrogen, USA) according to the manufacturer’s instructions. mRNA was reverse transcribed to cDNA using a PrimeScript RT Reagent Kit with gDNA Eraser (RR047A, Takara, Japan) according to the manufacturer’s instructions. Quantitative PCR (qPCR) was subsequently performed with BlazeTaq qPCR Mix (QP031-01, GeneCopoeia, USA). Expression was measured in triplicate in a Bio-Rad CFX Connect Real-Time System and normalised to human *ACTB* expression. The number of independent experiments was carried out three times. All the primer sequences that were used for qPCR are listed in Supplementary Table S1.

### Immunohistochemical (IHC), immunofluorescence (IF) and Western blotting (WB) analyses

The protocols of IHC and IF assays are described in greater detail in the supplementary materials. The product of the scores for intensity and proportion was used to signify the level of DAGLA expression. The immunoreactivity was reviewed and evaluated by two pathologists who were blinded to the clinical data. For protein extraction, cells were washed with ice-cold phosphate-buffered saline (PBS) and lysed on ice with radioimmunoprecipitation assay (RIPA) lysis buffer (R0020, Solarbio, China) for 30 min. The lysates were centrifuged at 13,800×*g* for 20 min, and the protein concentrations were measured with an Enhanced BCA Protein Assay Kit (P0010, Beyotime, China). Extracted proteins were separated by sodium dodecyl sulfate–polyacrylamide gel electrophoresis-page (SDS–PAGE). The number of independent experiments was carried out three times. The primary antibodies that were used are listed in Supplementary Table S2.

### Infection experiment

The lentiviral plasmids GV367-DAGLA and GV493-shDAGLA and the negative sequences were purchased from Genechem (Shanghai, China). Lentiviral constructs expressing short hairpin RNAs (shRNAs) and CMV promotor sequences were purchased from GeneChem (Shanghai, China) and were used for stable knockdown and overexpression in HCC cells, respectively. After 48 h, infected cells were cultured in a medium supplemented with puromycin (5 μg/ml, Solarbio, China) for 1 week to identify clones with stable expression. The sequences of the shRNAs that were used are listed in Supplementary Table S3.

### EdU incorporation assay

The 5-ethynyl-20-deoxyuridine (EdU) incorporation assay was performed using an EdU assay kit (Beyotime Biotechnology). Briefly, HCC cells were seeded in 24-well plates at 1 × 10^5^ cells/well for 24 h. Then, 10 µM EdU reagent was added to each well and incubated for 2 h at 37 °C. Finally, the HCC cells were photographed using a fluorescence microscope (Nikon, Japan), and the percentage of EdU-positive cells was calculated.

### ELISA

Cell and tissue samples were separately sonicated and homogenised in PBS, followed by centrifugation to obtain the supernatants. The lysate supernatants were analysed immediately or aliquoted and stored at 80 °C. The concentrations of 2-AG in the supernatants were measured using a commercial Human 2-Arachidonoylglycerol ELISA Kit (abx258337, Abbexa, UK) according to the manufacturer’s protocol. The concentrations of 2-AG were calculated from a standard curve.

### Chromatin immunoprecipitation (ChIP) assay

ChIP was carried out by using a Magna ChIP A/G Chromatin Immunoprecipitation Kit (17-10085, Millipore, USA) according to the manufacturer’s instructions. In brief, we fixed 1 × 10^7^ HCC cells in 37% formaldehyde for 10 min at ambient temperature. The fixed cells were harvested, lysed, and sonicated for 20 cycles of 10 s ON/30 s OFF using Bioruptor Pico (Bioruptor, Belgium). Antibodies against TEAD2 and rabbit IgG (Cell Signaling Technology, USA) were used for immunoprecipitation. PCR amplification of the precipitated DNA was performed. The number of independent experiments was carried out three times. The specific methods and primer sequences used for ChIP–qPCR were listed in the supplementary materials.

### RNA sequencing (RNA-seq) and analysis of RNA-seq data

RNA-seq and data analysis were performed as previously described [4]. Briefly, total mRNA was extracted from HCC cells by a spin column kit (Fastagen Biotech, China) and evaluated by an RNA 6000 kit (Agilent Technologies, Santa Clara, CA). Then, RNA libraries were established with a TruSeq RNA library preparation kit (Illumina, San Diego, CA), quantified by quantitative real-time PCR, normalised, pooled, and sequenced using an Illumina HiSeq 4000 instrument. Statistical analysis and ggplot2 (v3.3.2) were completed using R program v4.0.3, and a *P*-value < 0.05 was considered statistically significant. The accession number of RNA-seq raw data in the GEO database is GSE230174.

### Bioinformatics databases and statistical analysis

RNA-sequencing expression profiles and corresponding clinical information of HCC patients were obtained from the TCGA database (https://portal.gdc.com) and GTEx database (https://gtexportal.org/). Corresponding statistical analysis and ggplot2 (v3.3.2) were completed using R program v4.0.3, and a *P*-value < 0.05 was considered statistically significant. All independent experiments were carried out three times, and corresponding statistical analyses of the experimental results were performed with IBM SPSS Statistics 20.0 and GraphPad Prism 8 software. The variances are similar between the groups undergoing statistically compared. The values are presented as the means ± standard deviations (SDs). Student’s *t*-test was used for comparisons between groups, and the chi-square test was used for comparisons of categorical variables. Overall survival (OS) and recurrence-free survival (RFS) were evaluated by using the Kaplan–Meier method and compared by using the log-rank test. Multivariate Cox proportional hazards regression analysis was used to identify the independent prognostic factors. All tests were two-tailed, and *P*-values < 0.05 were considered statistically significant.

## Supplementary information

Supplementary figure 1

Supplementary figure 2

Supplementary figure 3

Supplementary figure 4

Supplementary figure 5

Supplementary figure 6

Supplementary figure legends

Supplementary Tables

Supplementary methods

Reproducibility checklist

Original western blots

## Data Availability

The data that support the findings of this study are available in the supplementary material of this article. The original western blot data has also been placed in the supplementary material.
