# Supplementary figures and images for: Diacylglycerol lipase alpha promotes hepatocellular carcinoma progression and induces lenvatinib resistance by enhancing YAP activity
Source: Cell Death Dis. 2023 Jul 6;14(7):404. doi: 10.1038/s41419-023-05919-5 (PMC10325985; doi:10.1038/s41419-023-05919-5)

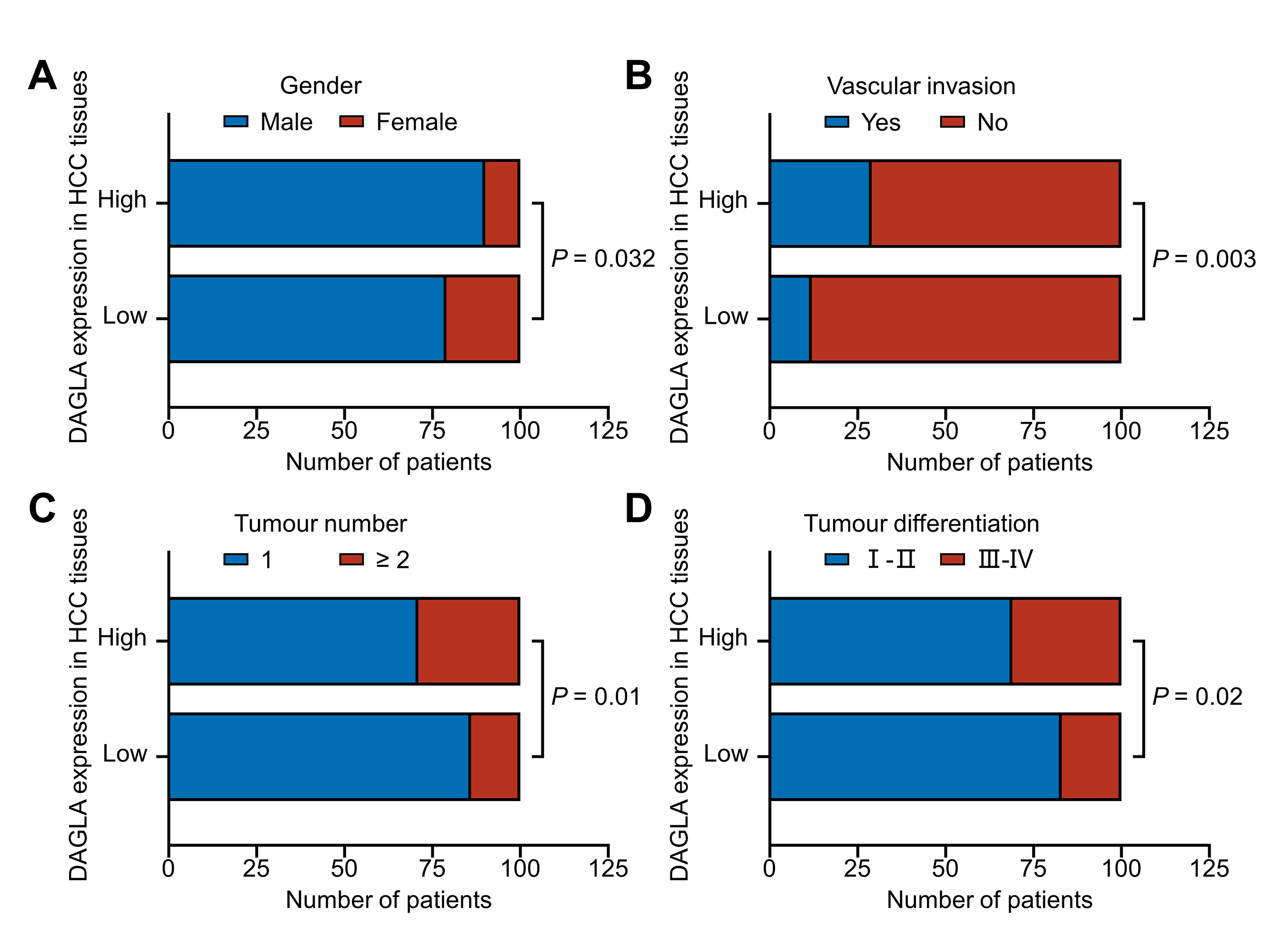

Supplement: Supplementary file 1 — Supplementary figure 1 [file 41419_2023_5919_MOESM1_ESM.tif]

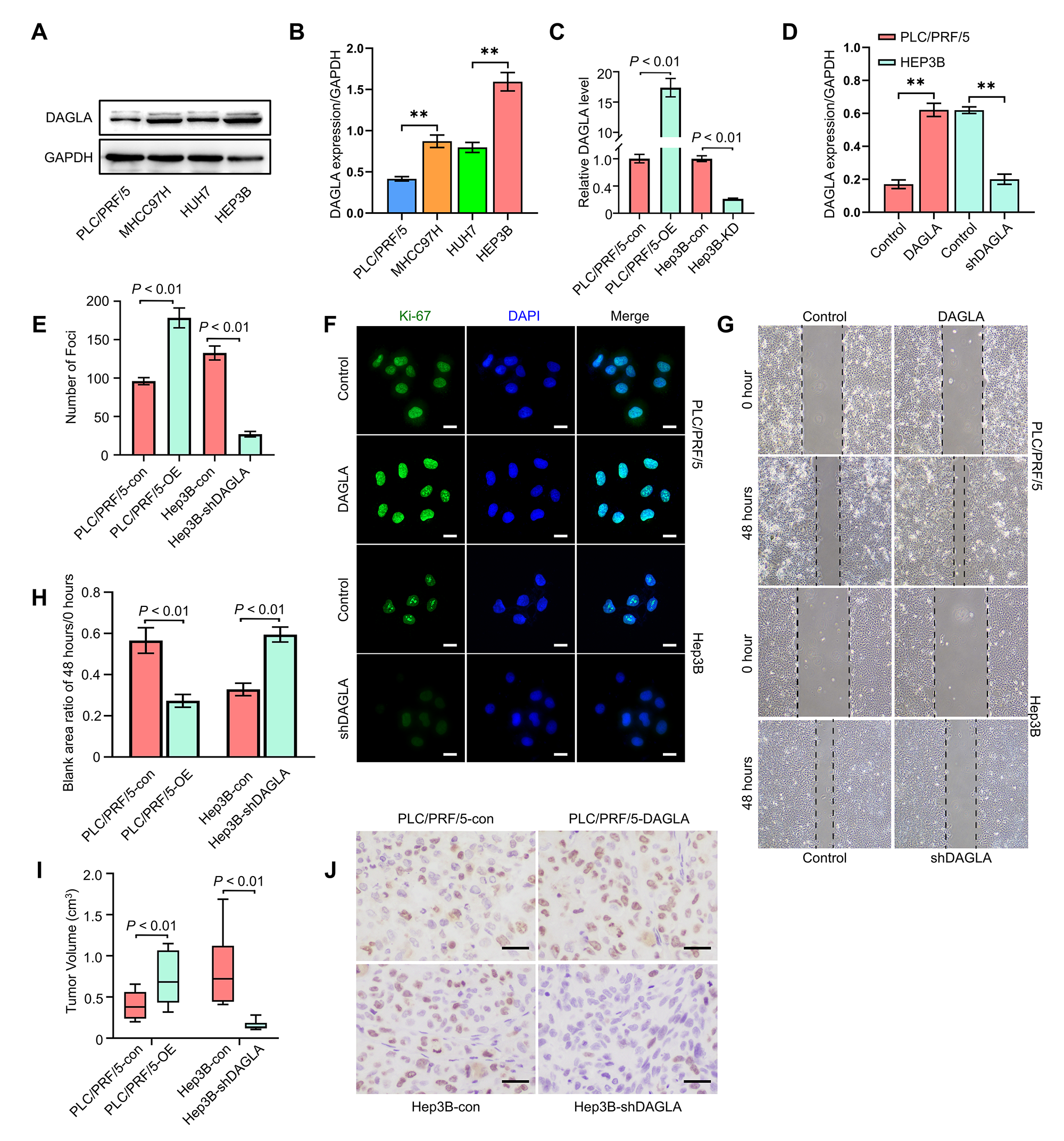

Supplement: Supplementary file 2 — Supplementary figure 2 [file 41419_2023_5919_MOESM2_ESM.tif]

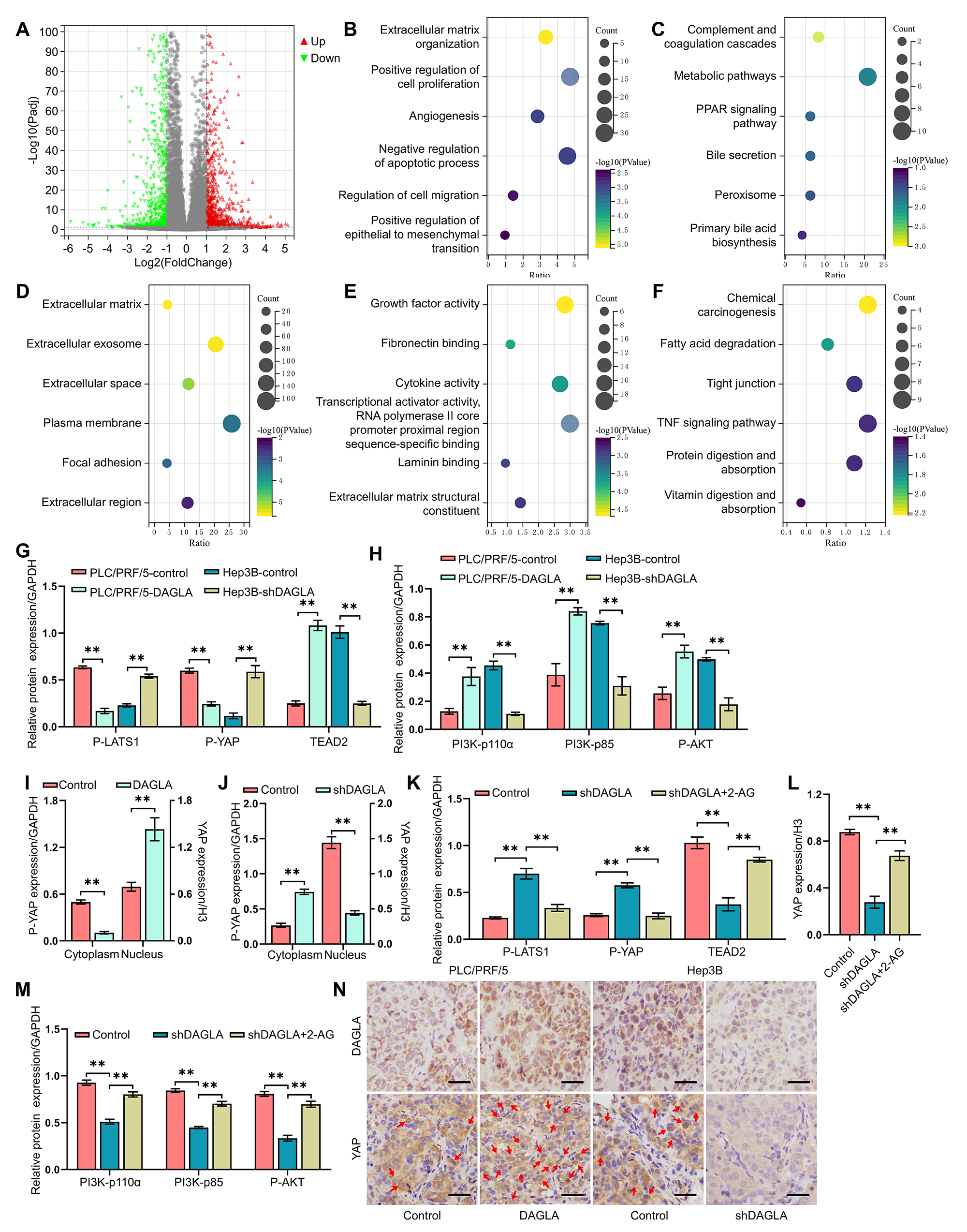

Supplement: Supplementary file 3 — Supplementary figure 3 [file 41419_2023_5919_MOESM3_ESM.tif]

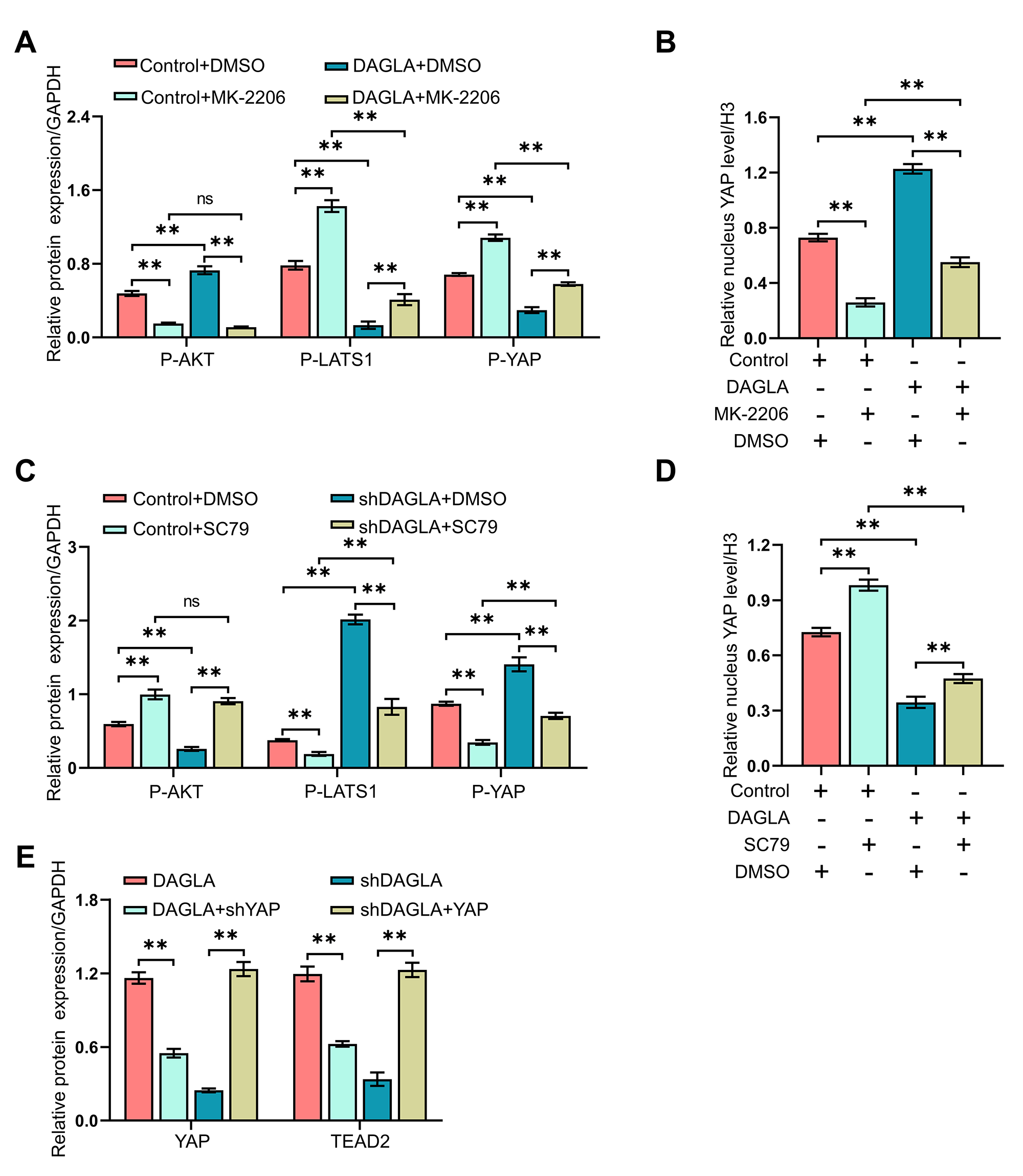

Supplement: Supplementary file 4 — Supplementary figure 4 [file 41419_2023_5919_MOESM4_ESM.tif]

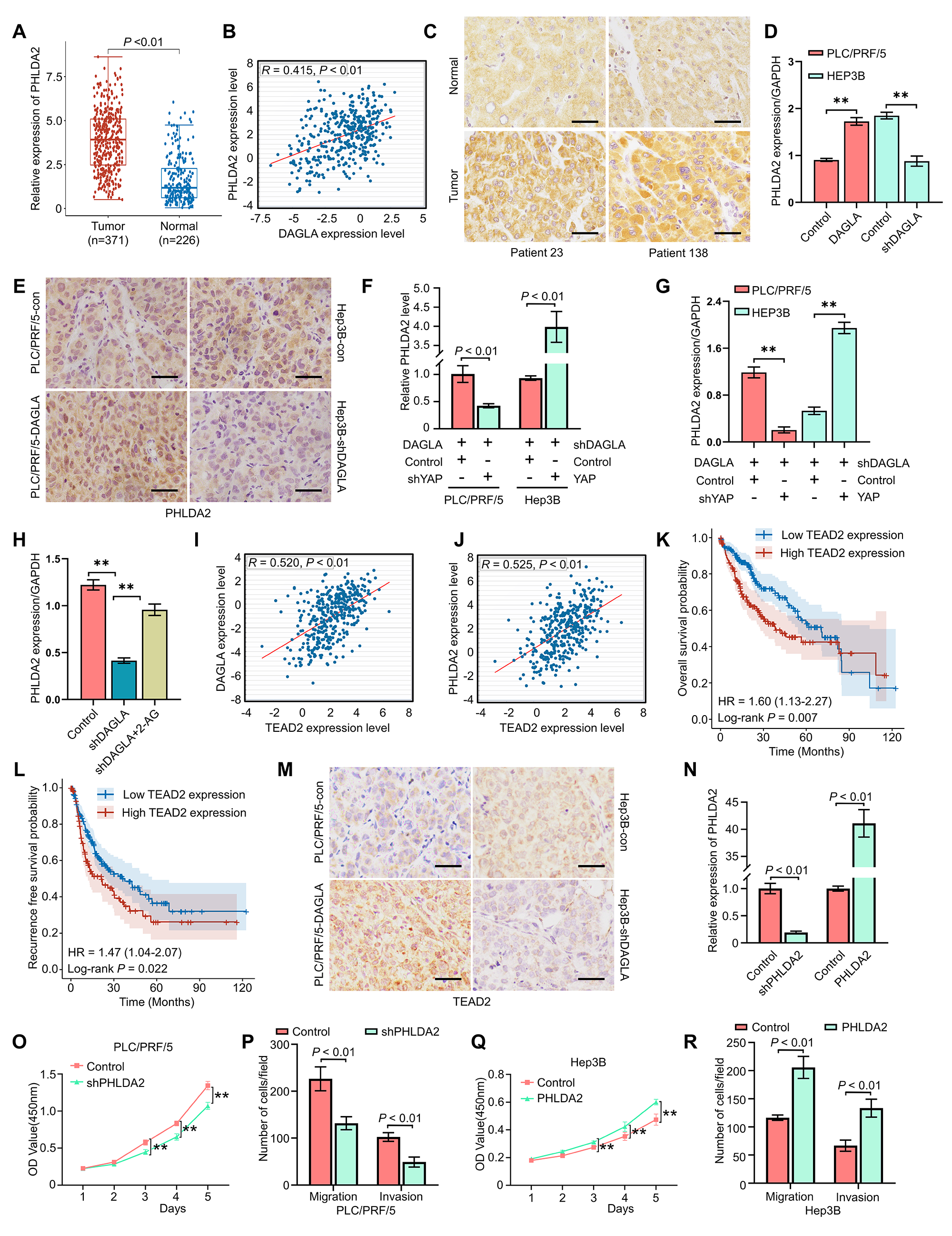

Supplement: Supplementary file 5 — Supplementary figure 5 [file 41419_2023_5919_MOESM5_ESM.tif]

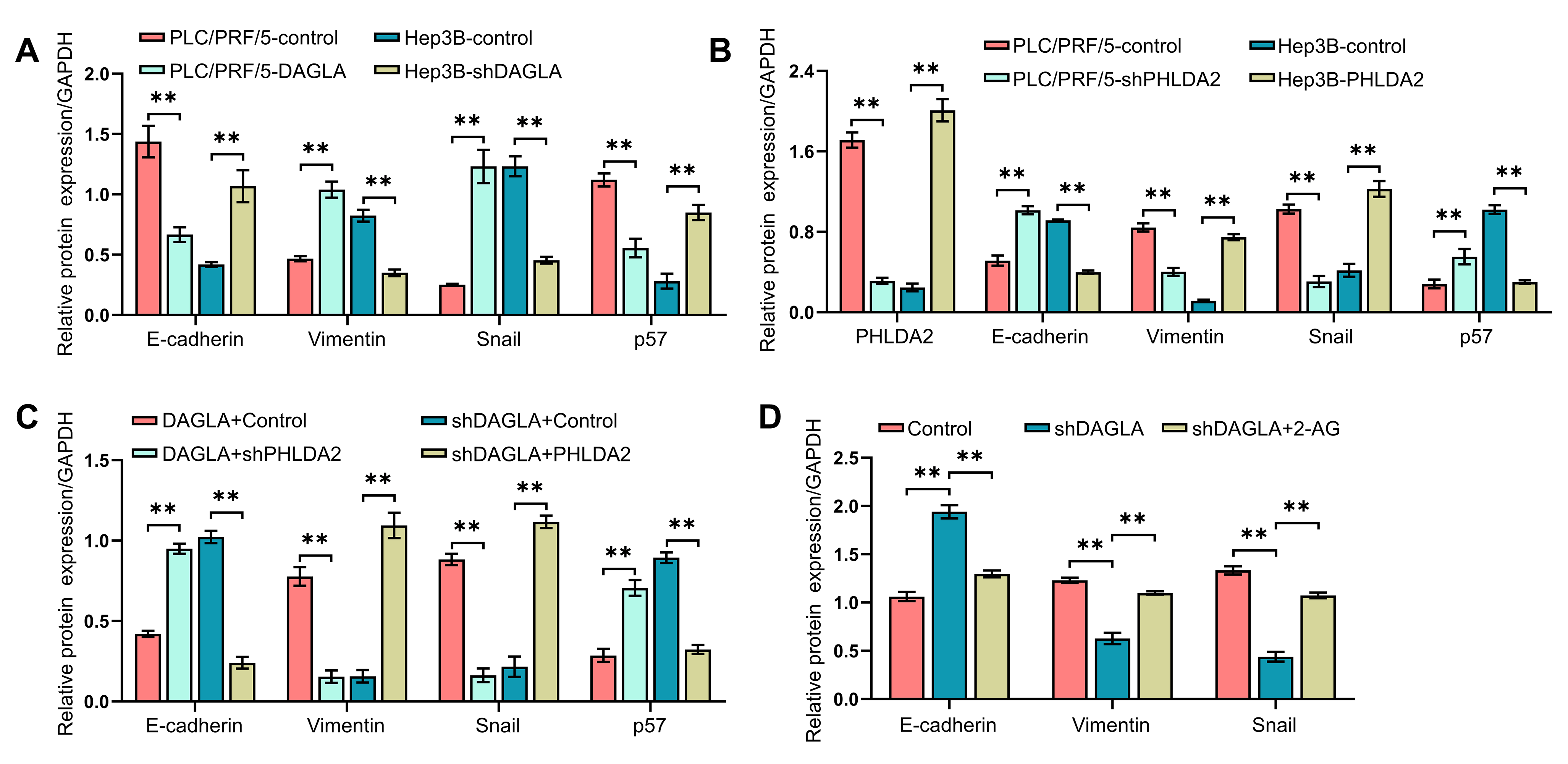

Supplement: Supplementary file 6 — Supplementary figure 6 [file 41419_2023_5919_MOESM6_ESM.tif]
