# Supplementary material for: Diacylglycerol lipase alpha promotes hepatocellular carcinoma progression and induces lenvatinib resistance by enhancing YAP activity
Source: Cell Death Dis. 2023 Jul 6;14(7):404. doi: 10.1038/s41419-023-05919-5 (PMC10325985; doi:10.1038/s41419-023-05919-5)
Supplement: Supplementary file 7 — Supplementary figure legends [file 41419_2023_5919_MOESM7_ESM.docx]

**Supplementary figure legends**

**Figure S1.** (A-D) The distribution of gender, vascular invasion, tumour number and tumour differentiation in high DAGLA group and low DAGLA group in HCC patients.

**Figure S2.** (A, B) WB were used to determine the basal DAGLA mRNA level in the PLC/PRF/5, MHCC97H, HUH7 and Hep3B cell lines. (C) qRT–PCR results confirmed the OE and KD efficiencies of DAGLA. (D) The statistical analysis of gray value of Fig. 2A. (E) Statistical analysis of colony counts. (F) IF staining for Ki-67 revealed the effect of DAGLA on HCC cell proliferation. Scale bars, 10 μm. (G, H) Wound healing assays were used to evaluate the migration ability of DAGLA-OE and DAGLA-KD cells. Magnification, 100×. (I) The statistical analysis of the volume of xenograft tumors. (J) The expression of Ki-67 in mouse xenograft tumours was detected by IHC staining. Scale bars, 50 μm.

**Figure S3.** (A) Volcano plot of DEGs from RNA-seq data. (B) Biological process of GO analysis revealed the potential mechanism through which DAGLA regulated HCC progression. (C) KEGG pathway enrichment analysis of the upregulated DEGs showed the enriched signalling pathways in HCC cells. (D, E) GO enrichment analysis of cellular components and molecular functions indicated the possible site at which and mechanism by which DAGLA regulates HCC progression. (F) KEGG pathway enrichment analysis of negatively correlated genes with DAGLA in TCGA database showed the enriched signalling pathways correlated with DAGLA in HCC. (G-M) The statistical analysis of gray value of Fig. 3F-I, K-M, respectively. (N) The expression of DAGLA and YAP in mouse xenograft tumours was detected by IHC staining. Scale bars, 50 μm.

**Figure S4.** (A, B) The statistical analysis of gray value of Fig. 4C, E. (C, D) The statistical analysis of gray value of Fig. 4F, G. (E) The statistical analysis of gray value of Fig. 4M.

**Figure S5.**  (A) Analysis of TCGA and GTEx databases showed that PHLDA2 was upregulated in HCC tissues compared with normal tissues. (B) The correlation between DAGLA and PHLDA2 expression was analysed from TCGA database. (C) IHC indicated that PHLDA2 expression in HCC tissues was significantly higher than that in adjacent non-tumour tissues. Scale bars, 50 μm. (D) The statistical analysis of gray value of Fig. 5H. (E) The PHLDA2 expression in mouse xenograft tumours was detected by IHC staining. Scale bars, 50 μm. (F) qRT–PCR showed the expression of PHLDA2 in Hep3B-shDAGLA+YAP-OE cells and PLC/PRF/5-DAGLA+YAP-KD cells. (G, H) The statistical analysis of gray value of Fig. 5I, J. (I) The correlation between DAGLA and TEAD2 expression was analysed from TCGA database. (J) TCGA database revealed a significant positive correlation between PHLDA2 and TEAD2 expression. (K, L) TCGA database revealed that high TEAD2 level was correlated with worse OS and RFS in HCC patients. (M) The TEAD2 expression in mouse xenograft tumours was detected by IHC staining. Scale bars, 50 μm. (N) qRT–PCR confirmed the lentiviral transduction efficiency of PHLDA2 constructs. (O-R) CCK-8 and Transwell assays verified the effect of PHLDA2 on HCC cell proliferation, migration and invasion abilities. ** *P* < 0.01.

**Figure S6.** (A, B) The statistical analysis of gray value of Fig. 6F, G. (C, D) The statistical analysis of gray value of Fig. 6H, I.
