# Supplementary material for: Diacylglycerol lipase alpha promotes hepatocellular carcinoma progression and induces lenvatinib resistance by enhancing YAP activity
Source: Cell Death Dis. 2023 Jul 6;14(7):404. doi: 10.1038/s41419-023-05919-5 (PMC10325985; doi:10.1038/s41419-023-05919-5)
Supplement: Supplementary file 8 — Supplementary Tables [file 41419_2023_5919_MOESM8_ESM.docx]

**Supplementary Table S1 List of primer sequences**

| Gene | Forward primer sequence | Reverse primer sequence |
| --- | --- | --- |
| β-actin | CATGTACGTTGCTATCCAGGC | CTCCTTAATGTCACGCACGAT |
| DAGLA | GACCCTCAAGTGCTTTGCCT | CAGAACCACAGCAGTCACGA |
| YAP | CTGACCCCCACTGGAGTAGT | TTTGCCATCTCCCAACCTGC |
| PHLDA2 | GACAGCCTCTTCCAGCTATGG | TCGATCTCCTTGTGGTCGGT |
| CDKN1C | CCACGATGGAGCGTCTTGT | GTCGTAATCCCAGCGGTTCT |
| PHLDA2 for ChIP | GATAGGAGCTCGGTCCGCTA | CCCCCTTCCTTACTCCCGA |

**Supplementary Table S2 List of primary and secondary antibodies**

| Antibody name | Corporation | Application |
| --- | --- | --- |
| Rabbit anti-human GAPDH | Abmart | WB:1/5000 |
| Goat anti-human DAGLA | Abcam | WB:1/1000 |
| Rabbit anti-human DAGLA | Merck | IHC:1/200 |
| Mouse anti-human Ki-67 | Cell Signaling Technology | IHC:1/400  IF:1/200 |
| Rabbit anti-human LATS1 | Cell Signaling Technology | WB:1/1000 |
| Rabbit anti-human p-LATS1 | Cell Signaling Technology | WB:1/1000 |
| Rabbit anti-human YAP | Cell Signaling Technology | WB:1/1000  IHC:1/250  IF:1/250 |
| Rabbit anti-human p-YAP | Cell Signaling Technology | WB:1/1000 |
| Rabbit anti-human TEAD2 | Huabio | WB:1/1000  ChIP:1/10 |
| Rabbit anti-human TEAD4 | Abcam | WB:1/1000 |
| Rabbit anti-human PI3K p110α | Cell Signaling Technology | WB:1/1000 |
| Rabbit anti-human PI3K p85 | Cell Signaling Technology | WB:1/1000 |
| Rabbit anti-human AKT | Cell Signaling Technology | WB:1/1000 |
| Rabbit anti-human p-AKT | Cell Signaling Technology | WB:1/1000 |
| Rabbit anti-human GSK3 | Affinity | WB:1/1000 |
| Rabbit anti-human Histone H3 | Abcam | WB:1/1000 |
| Rabbit anti-human PHLDA2 | Proteintech | WB:1/1000  IHC:1/200 |
| Rabbit anti-human snail | Cell Signaling Technology | WB:1/1000 |
| Mouse anti-human vimentin | Abcam | WB:1/2000 |
| Mouse anti-human E-cadherin | Cell Signaling Technology | WB:1/1000  IHC:1/100  IF:1/100 |
| Rabbit anti-human p57 | Cell Signaling Technology | WB:1/1000 |
| Alexa Fluor 488 donkey anti-mouse IgG | Thermo Fisher Technology | IF:1/1000 |
| Alexa Fluor 555 donkey anti-rabbit IgG | Thermo Fisher Technology | IF:1/1000 |
| HRP-labeled goat anti-rabbit IgG | Origene | WB:1/2500 |
| HRP-labeled goat anti-mouse IgG | Origene | WB:1/2500 |
| HRP-labeled rabbit anti-goat IgG | Origene | WB:1/2500 |
| Normal rabbit IgG | Cell Signaling Technology | ChIP:1μg/well |

**Supplementary Table S3 List of transfection sequences**

| Gene | Transfection sequences |
| --- | --- |
| shDAGLA | GCGACAACAAGGCCTTCAA |
| shYAP | GAGATGGAATGAACATAGA |
| shPHLDA2 | ACCACAAGGAGATCGACTT |

**Supplementary Table S4 Statistics for DAGLA and clinicopathologic features in HCC patients**

| Clinicopathological feature | DAGLA expression | | | *χ2* | *P* value |
| --- | --- | --- | --- | --- | --- |
|  | Total | Low | High |  |  |
|  | 200 | 100 | 100 |  |  |
| Age (years) | | | | | |
| ≤50 | 95 | 48 | 47 | 0.020 | 0.887 |
| >50 | 105 | 52 | 53 |  |  |
| Gender | | | | | |
| Male | 169 | 79 | 90 | 4.619 | 0.032 |
| Female | 31 | 21 | 10 |  |  |
| Tumour diameter | | | | | |
| ≤5 cm | 133 | 70 | 63 | 1.100 | 0.294 |
| >5 cm | 67 | 30 | 37 |  |  |
| Vascular invasion | | | | | |
| No | 159 | 88 | 71 | 8.866 | 0.003 |
| Yes | 41 | 12 | 29 |  |  |
| Tumour capsule | | | | | |
| Yes | 71 | 33 | 38 | 0.546 | 0.460 |
| None | 129 | 67 | 62 |  |  |
| Tumour number | | | | | |
| 1 | 157 | 86 | 71 | 6.666 | 0.010 |
| ≥2 | 43 | 14 | 29 |  |  |
| AFP | | | | | |
| ≤400 ng/ml | 74 | 37 | 37 | 0.000 | 1.000 |
| >400 ng/ml | 126 | 63 | 63 |  |  |
| Tumour differentiation | | | | | |
| I-II | 152 | 83 | 69 | 5.373 | 0.020 |
| III-IV | 48 | 17 | 31 |  |  |
| Liver cirrhosis | | | | | |
| None | 34 | 21 | 13 | 2.268 | 0.132 |
| Yes | 166 | 79 | 87 |  |  |
| HBsAg | | | | | |
| Negative | 41 | 26 | 15 | 3.712 | 0.054 |
| Positive | 159 | 74 | 85 |  |  |

**Supplementary Table S5 Univariate and multivariate analysis of OS in HCC patients**

| Variable | Univariate | | Multivariate | |
| --- | --- | --- | --- | --- |
|  | χ2 | Pvalue | HR (95%Cl) | P value |
| DAGLA level (high vs low) | 19.146 | <0.001 | 2.241 (1.400-3.587) | 0.001 |
| Age (years) (≤50 vs >50) | 0.722 | 0.395 | n.a. | n.a. |
| Gender (male vs female) | 0.148 | 0.701 | n.a. | n.a. |
| Tumour diameter (cm) (≤5 vs >5) | 1.338 | 0.247 | n.a. | n.a. |
| Vascular invasion (yes vs no) | 8.596 | 0.003 | 1.297 (0.779-2.159) | 0.318 |
| Tumour capsule (none vs yes) | 1.866 | 0.172 | n.a. | n.a. |
| Tumour number (≥2 vs 1) | 12.678 | <0.001 | 1.595 (0.982-2.592) | 0.059 |
| AFP (ng/ml) (≤400 vs >400) | 5.018 | 0.025 | 1.493 (0.916-2.434) | 0.108 |
| Tumour differentiation (III-IV vs I-II) | 0.434 | 0.501 | n.a. | n.a. |
| Liver cirrhosis (yes vs none) | 4.463 | 0.035 | 1.717 (0.855-3.448) | 0.129 |
| HBsAg (positive vs negative) | 1.527 | 0.217 | n.a. | n.a. |

n.a. not applicable.

**Supplementary Table S6 Univariate and multivariate analysis of RFS in HCC patients**

| Variable | Univariate | | Multivariate | |
| --- | --- | --- | --- | --- |
|  | χ2 | Pvalue | HR(95%Cl) | P value |
| DAGLA level (high vs low) | 24.732 | <0.001 | 2.385 (1.592-3.575) | <0.001 |
| Age (years) (≤50 vs >50) | 1.020 | 0.313 | n.a. | n.a. |
| Gender (male vs female) | 0.002 | 0.968 | n.a. | n.a. |
| Tumour diameter (cm) (≤5 vs >5) | 0.130 | 0.719 | n.a. | n.a. |
| Vascular invasion (yes vs no) | 2.707 | 0.100 | n.a. | n.a. |
| Tumour capsule (none vs yes) | 1.744 | 0.187 | n.a. | n.a. |
| Tumour number (≥2 vs 1) | 20.094 | <0.001 | 2.098 (1.380-3.190) | 0.001 |
| AFP (ng/ml) (≤400 vs >400) | 0.163 | 0.686 | n.a. | n.a. |
| Tumour differentiation (III-IV vs I-II) | 0.008 | 0.929 | n.a. | n.a. |
| Liver cirrhosis (yes vs none) | 3.090 | 0.079 | n.a. | n.a. |
| HBsAg (positive vs negative) | 2.053 | 0.152 | n.a. | n.a. |

n.a. not applicable.

**Supplementary Table S7 List of 33 common genes**

| Gene name | | |
| --- | --- | --- |
| **PHLDA2** | CDCA3 | ANXA2P2 |
| HPX | SSR3 | MAD2L1 |
| SΜLT1C2 | PSMD14 | GRK6 |
| C6orf47 | MTHFD1L | YWHAZ |
| SMOX | HJURP | TUBA1C |
| KCTD17 | ADM2 | SLC25A6 |
| PSMB5 | TNFRSF21 | CANT1 |
| NDRG3 | ENAH | B3GNT3 |
| KRT23 | FAM189B | MCM6 |
| CCDC86 | S100A11 | BGN |
| ZNF544 | RPN1 | ANXA2 |
