# Supplementary material for: Diacylglycerol lipase alpha promotes hepatocellular carcinoma progression and induces lenvatinib resistance by enhancing YAP activity
Source: Cell Death Dis. 2023 Jul 6;14(7):404. doi: 10.1038/s41419-023-05919-5 (PMC10325985; doi:10.1038/s41419-023-05919-5)
Supplement: Supplementary file 9 — Supplementary methods [file 41419_2023_5919_MOESM9_ESM.docx]

**Supplementary materials and methods**

**Immunohistochemical (IHC) staining**

Immunohistochemical staining was carried out on sections of the formalin-fixed samples on the tissue microarray. The slides were de-waxed by heating at 65 °C for 60 min followed by two washes with xylene. Tissues were then rehydrated, and endogenous peroxidase activity was blocked. Antigen retrieval was performed by heating the samples at 95 °C for 20 min in 1× ethylenediaminetetraacetic acid (EDTA, pH 8.0). Then, the TMA slides were placed in 2 N HCl for 30 min, rinsed in PBS and placed in 100 mM Tris–HCl (pH 8.5) for 10 min. They were then incubated in 5 % normal goat serum at room temperature for 60 min to reduce non-specific reactions. Subsequently, the slides were incubated overnight at 4 °C with primary antibody with appropriate concentration. Then, the slides were incubated with secondary antibody for 60 min at room temperature and stained with diaminobenzidine (DAB). Finally, the TMA slides were counterstained with haematoxylin, dehydrated and mounted with a coverslip using neutral gum. Phosphate buffer solution was used to replace the primary antibody as a negative control.

**Immunofluorescence (IF)**

The expression and location of targeted proteins in HCC cells were detected by immunofluorescence assay. In general, cells cultured on glass slides were gently washed by PBS and fixed by methanol for 15 minutes. Wash with PBS for 5 minutes, repeat 2 times. After treated with 1% TritonX-100 for 30 minutes, the fixed cells were blocked with bovine serum albumin for 60 minutes and incubated overnight directly in diluted primary antibody. The next day, after rinsing in PBS, the slices were incubated in Alexa Fluor secondary antibody with appropriate concentration for 60 minutes and were counterstained with DAPI (Solarbio, China) for 5 minutes. Finally, fluorescence was examined under fluorescent microscope (Nikon, Japan).
