# Supplementary material for: Diacylglycerol lipase alpha promotes hepatocellular carcinoma progression and induces lenvatinib resistance by enhancing YAP activity
Source: Cell Death Dis. 2023 Jul 6;14(7):404. doi: 10.1038/s41419-023-05919-5 (PMC10325985; doi:10.1038/s41419-023-05919-5)
Supplement: Supplementary file 11 — Original western blots [file 41419_2023_5919_MOESM11_ESM.pdf]

Original western blots

Figure 2A:

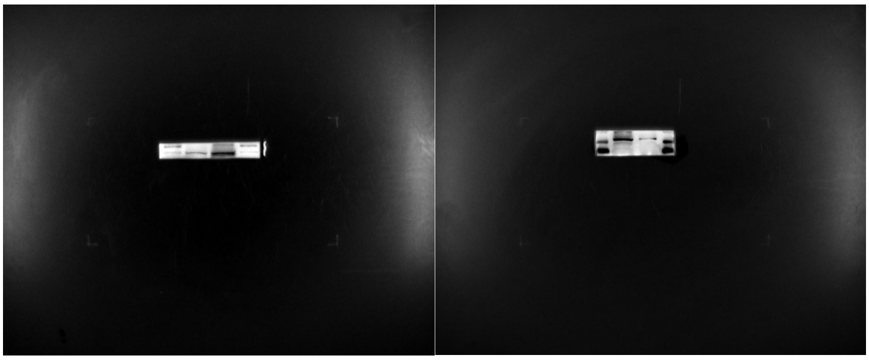

DAGLA 110KD

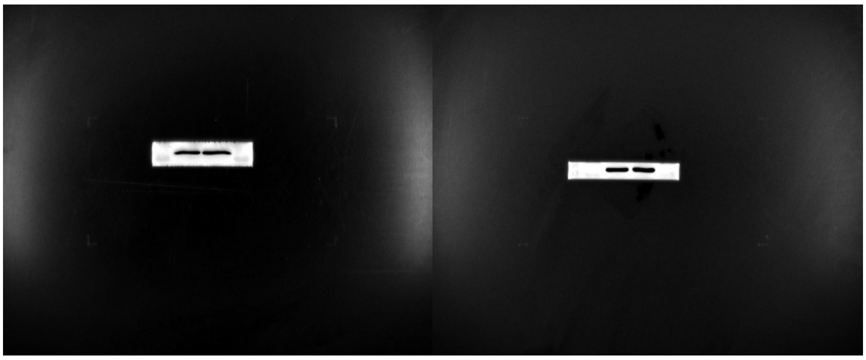

GAPDH 36KD

Figure S2B:

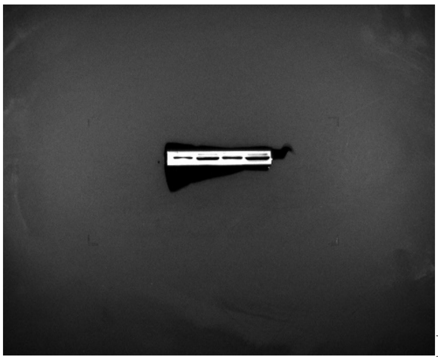

DAGLA 110KD

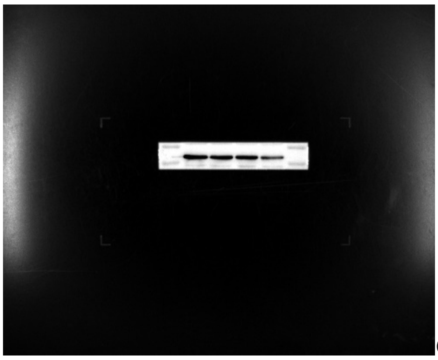

GAPDH 36KD

Figure 3F:

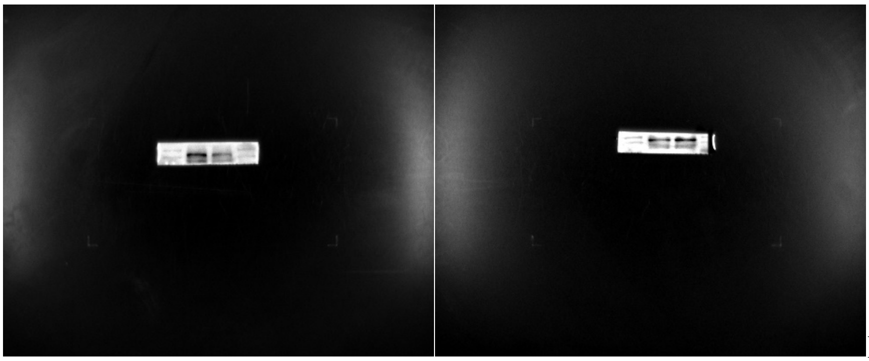

P-LATS1 140KD

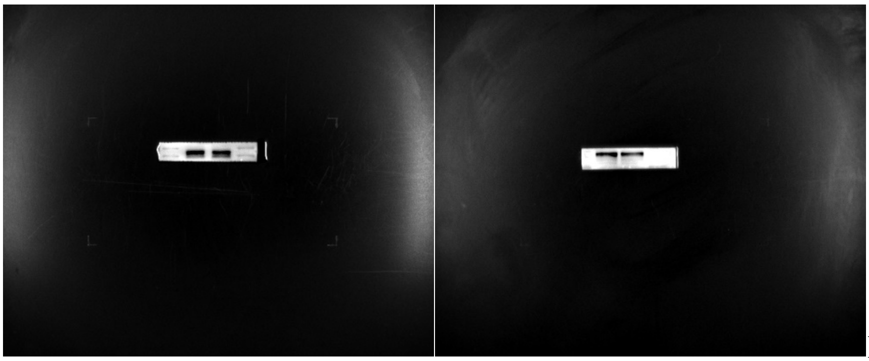

LATS1 140KD

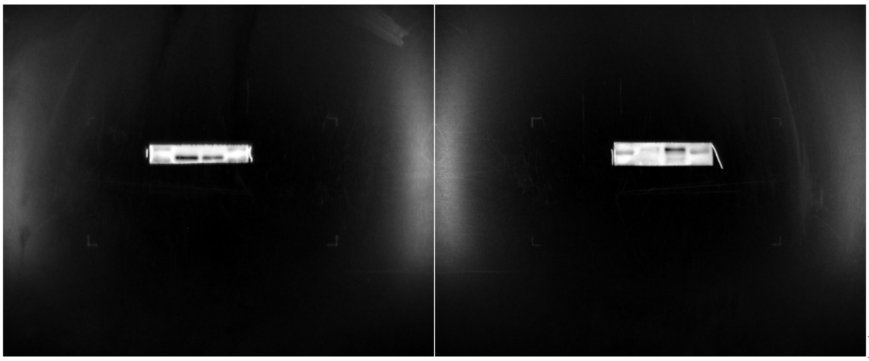

P-YAP 70KD

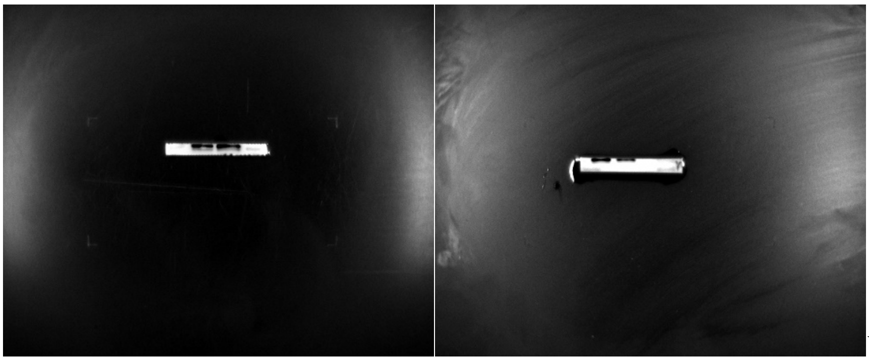

YAP 70KD

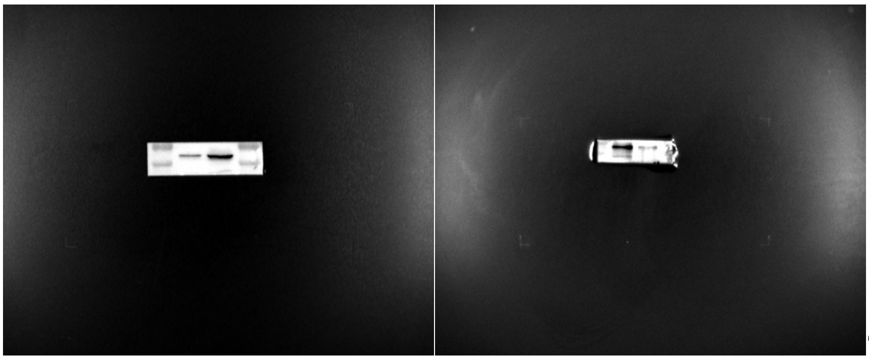

TEAD2 55KD

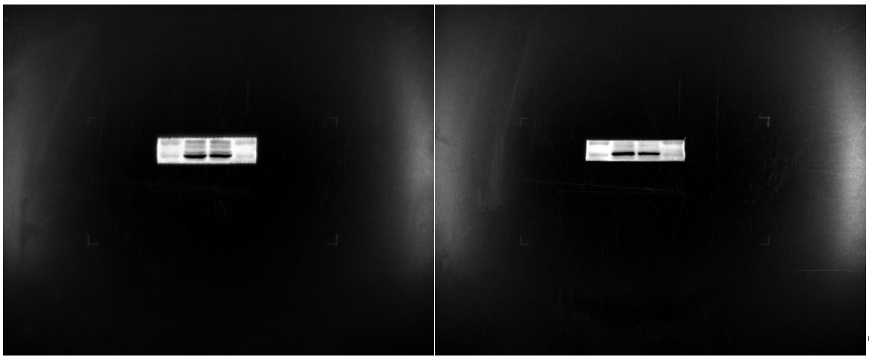

TEAD4 55KD

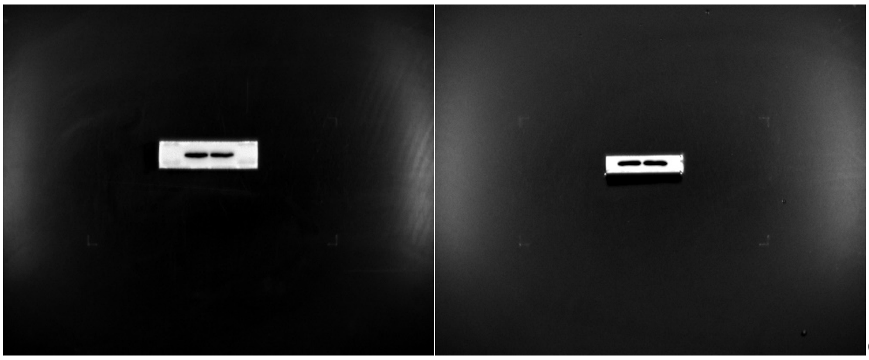

GAPDH 36KD

Figure 3G

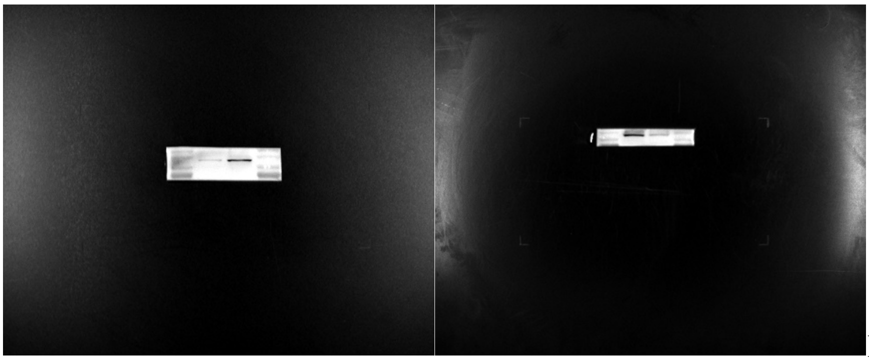

PI3Kp110 110KD

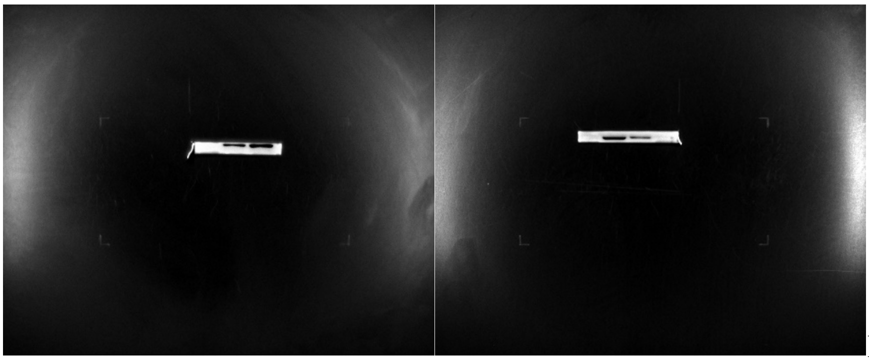
PI3K-p85 85KD

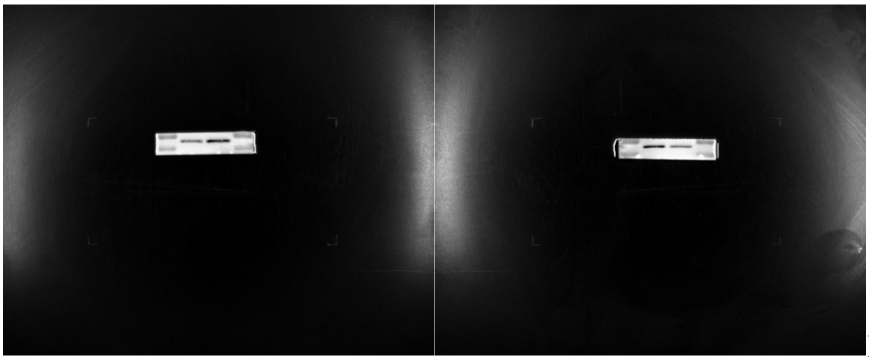
P-AKT 60KD

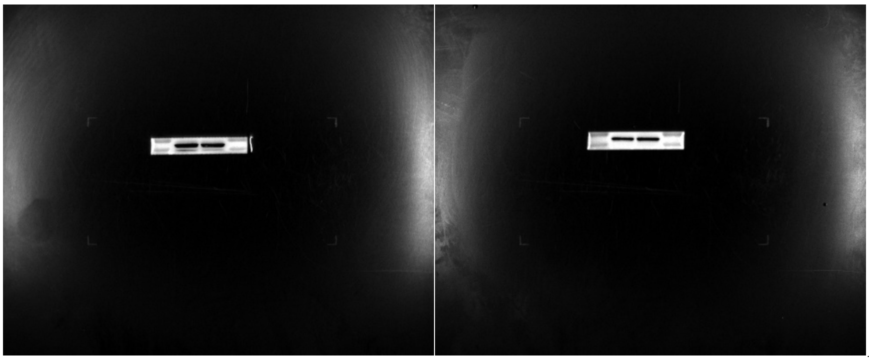
AKT 60KD

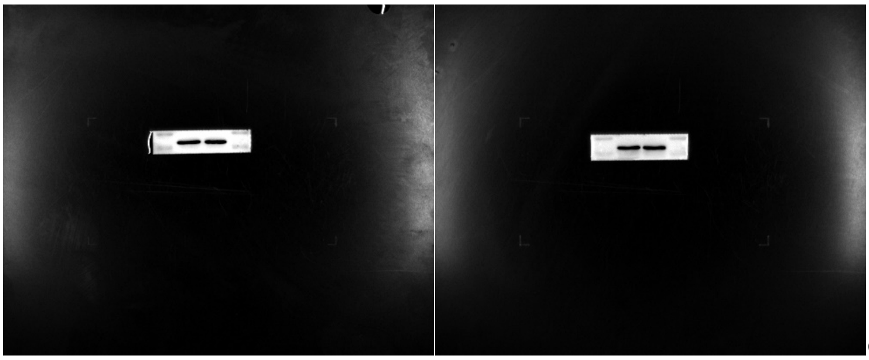
GAPDH 36KD

Figure 3H

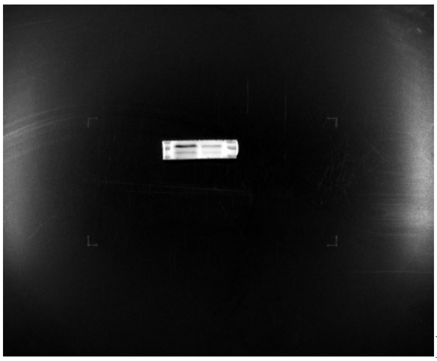
P-YAP 70KD

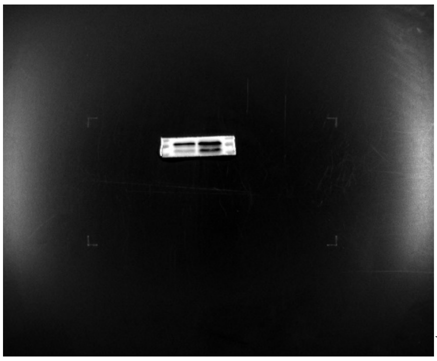
YAP 70KD

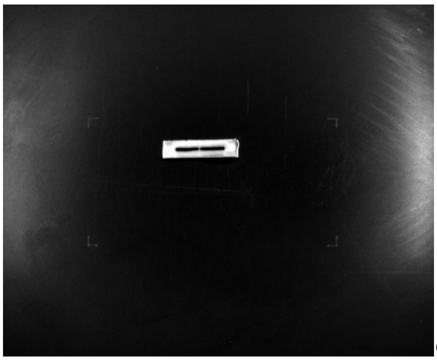
GAPDH 36KD

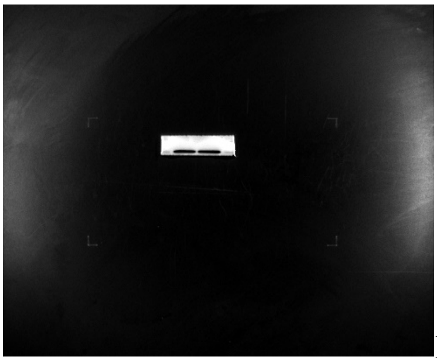
HistoneH3 17KD

Figure 3I

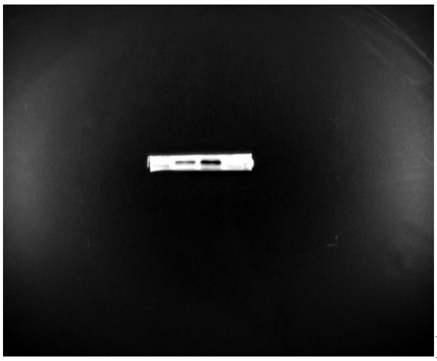
P-YAP 70KD

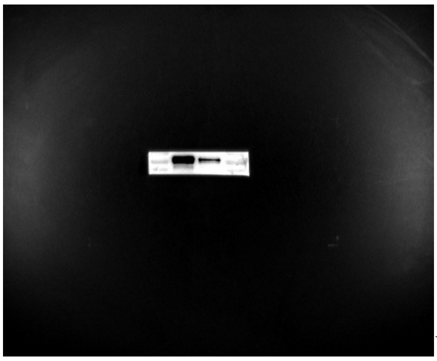
YAP 70KD

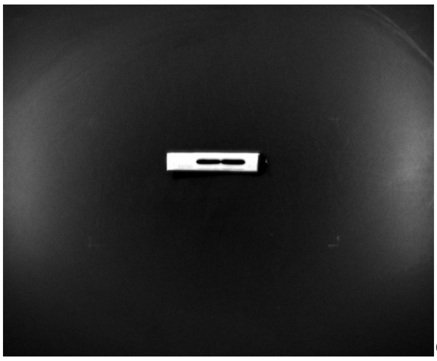
GAPDH 36KD

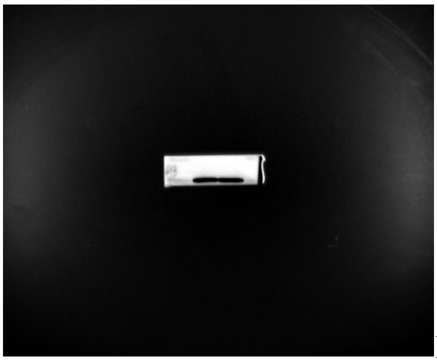
HistoneH3 17KD

Figure 3K

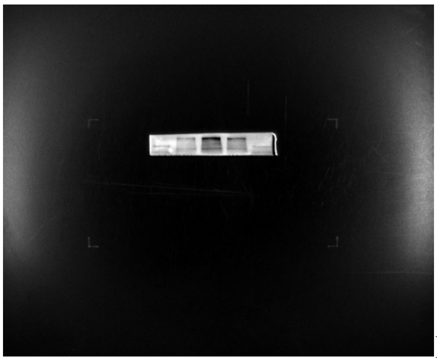

P-LATS1 140KD

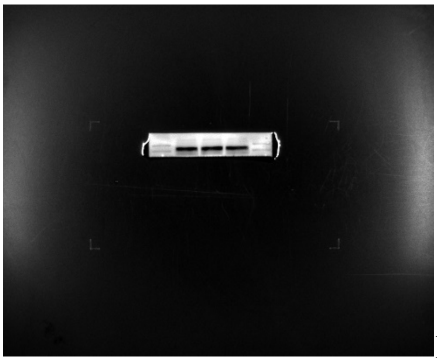

LATS1 140KD

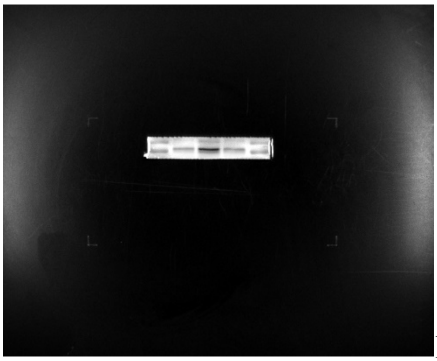

P-YAP 70KD

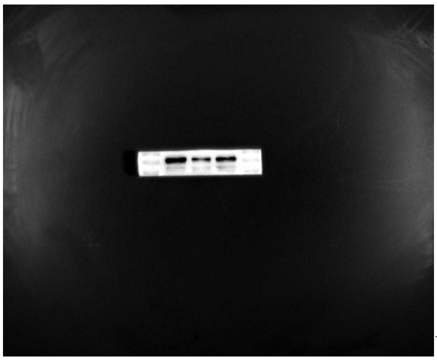

YAP 70KD

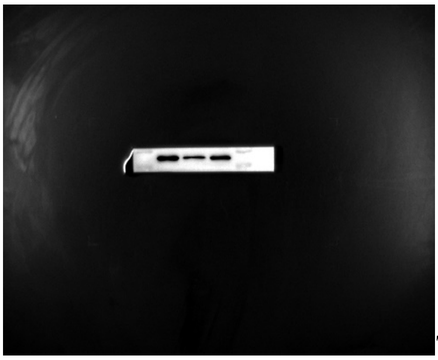

TEAD2 55KD

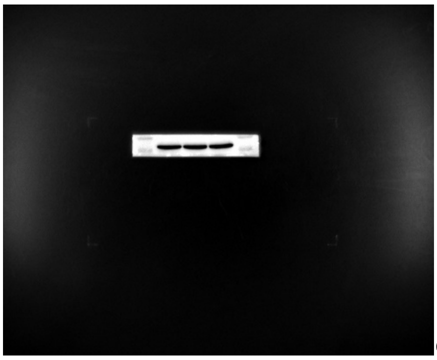

GAPDH 36KD

Figure 3L

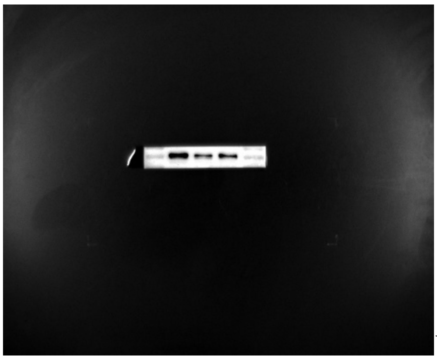

YAP 70KD

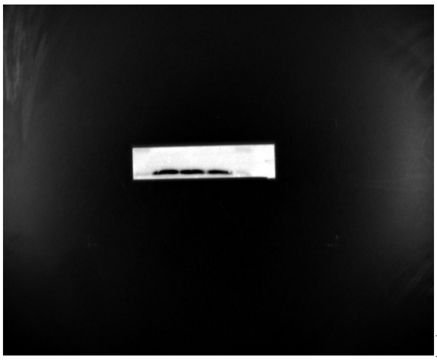

HistoneH3 17KD

Figure 3M

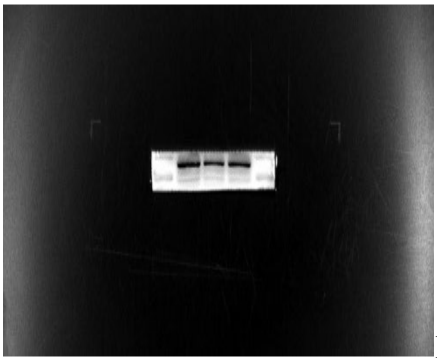

PI3Kp110 110KD

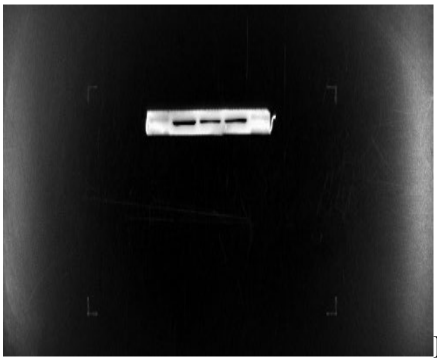

PI3K-p85 85KD

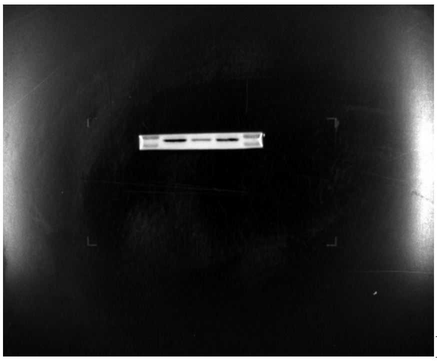

P-AKT 60KD

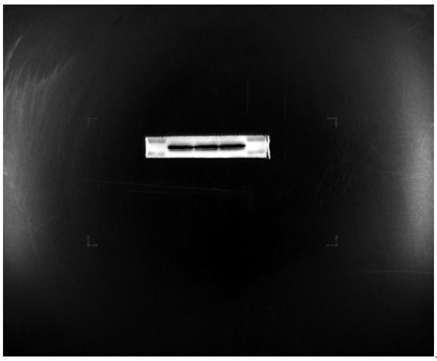

AKT 60KD

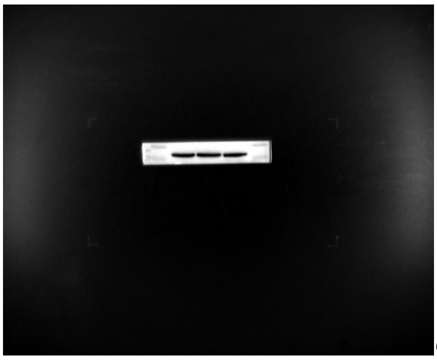
GAPDH 36KD

Figure 4C

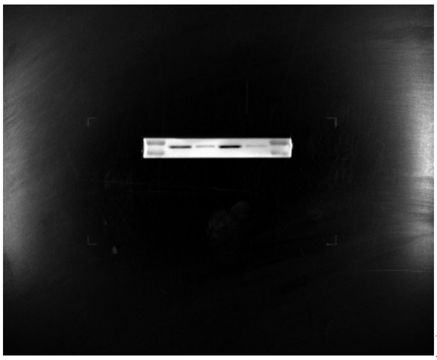
P-AKT 60KD

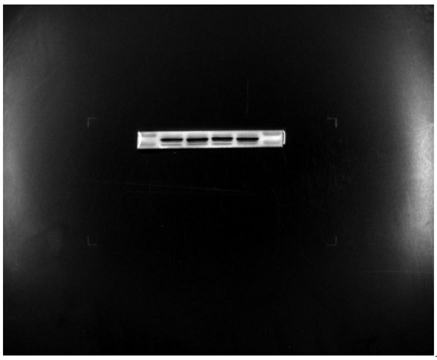
AKT 60KD

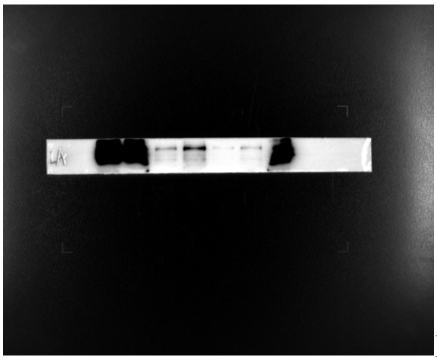
P-LATS1 140KD

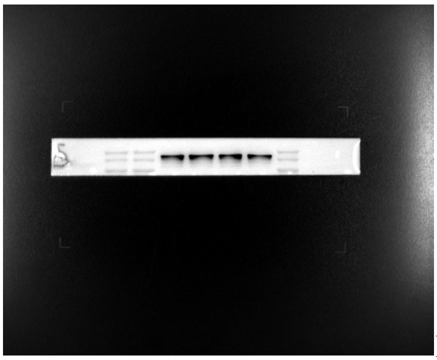
LATS1 140KD

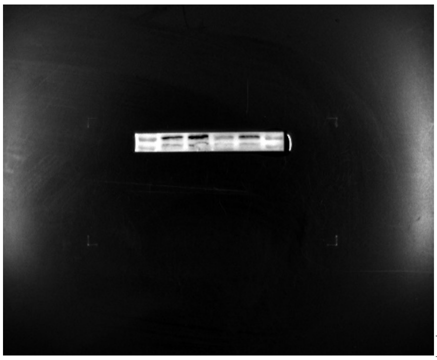
P-YAP 70KD

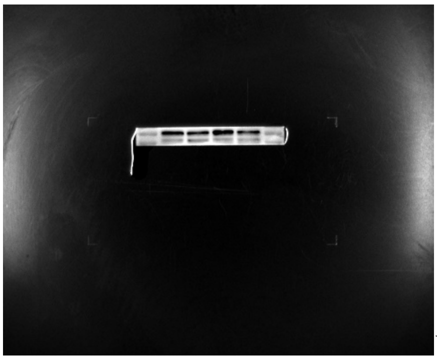
YAP 70KD

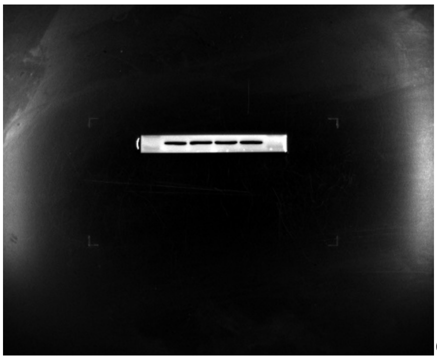
GAPDH 36KD

Figure 4E

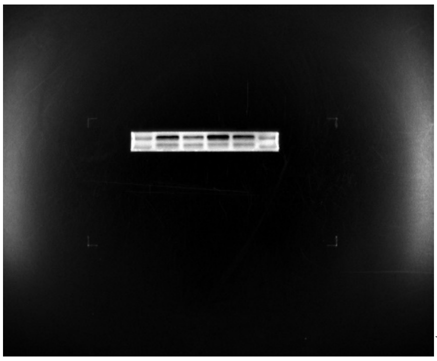

YAP 70KD

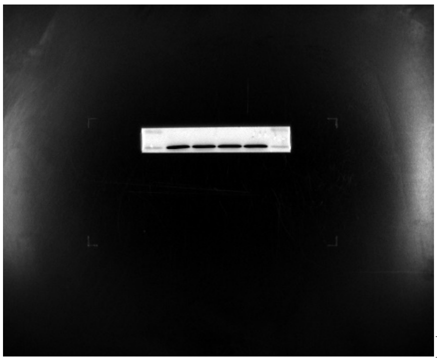

HistoneH3 17KD

Figure 4F

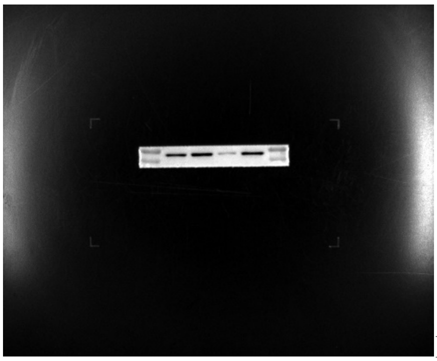

P-AKT 60KD

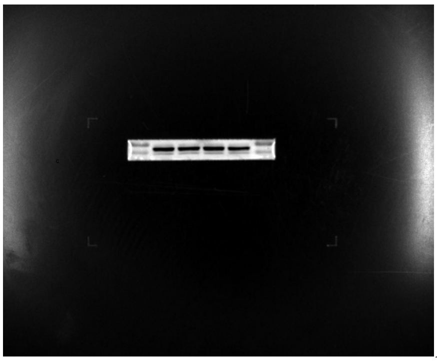

AKT 60KD

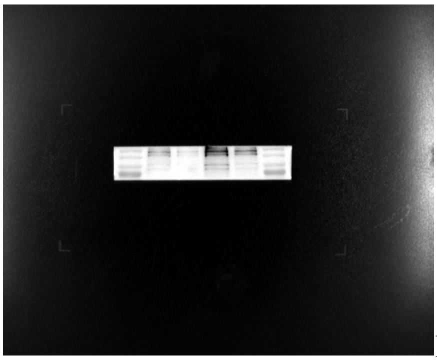
P-LATS1 140KD

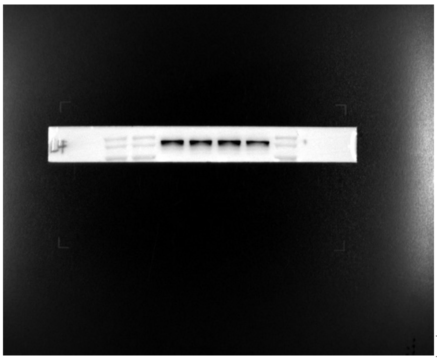
LATS1 140KD

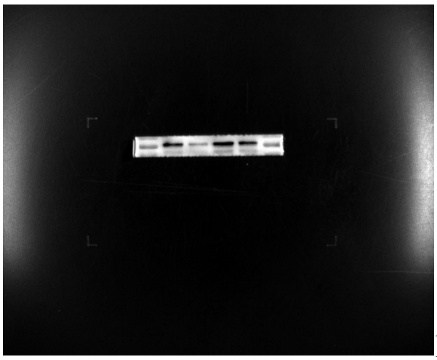
P-YAP 70KD

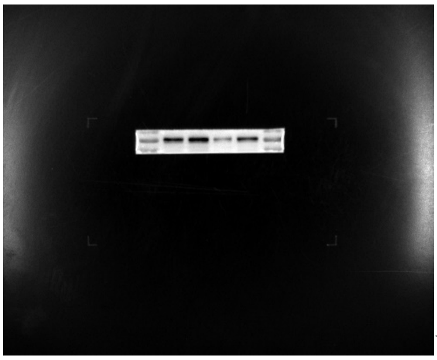
YAP 70KD

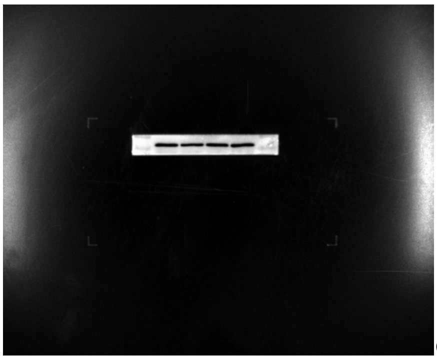

GAPDH 36KD

Figure 4G

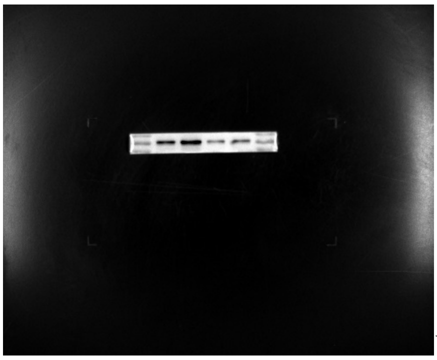

YAP 70KD

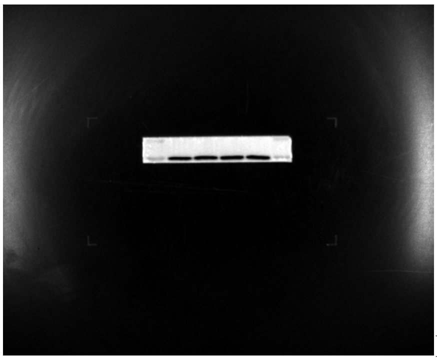

HistoneH3 17KD

Figure 4M

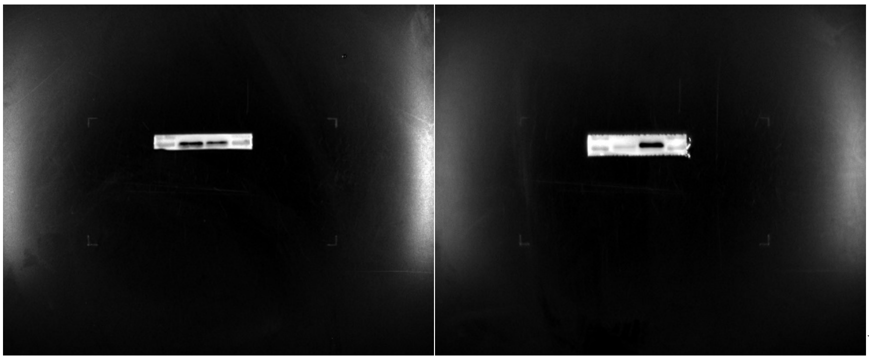

YAP 70KD

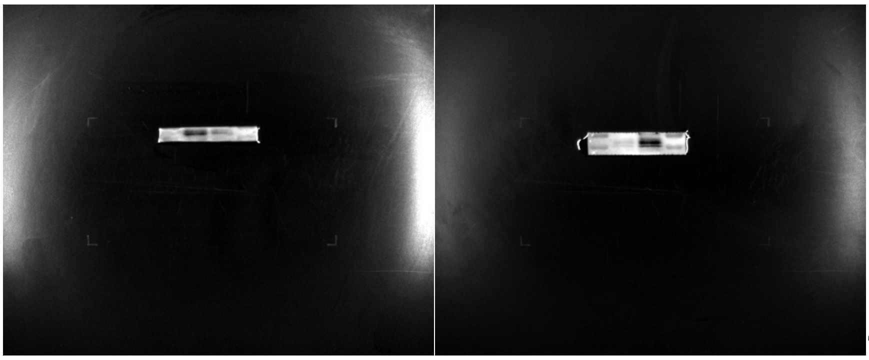

TEAD2 55KD

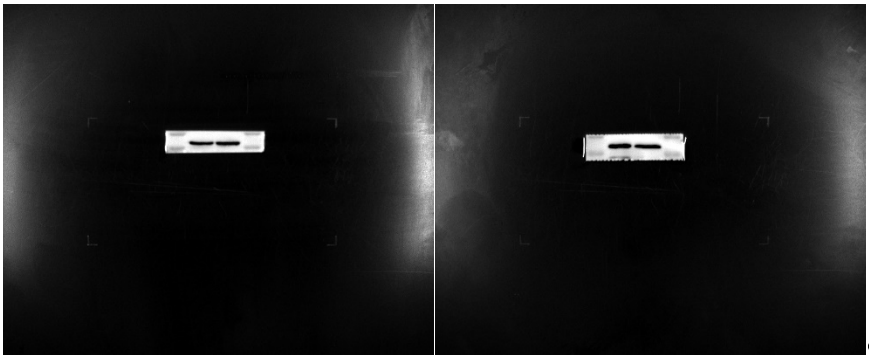

GAPDH 36KD

Figure 5H

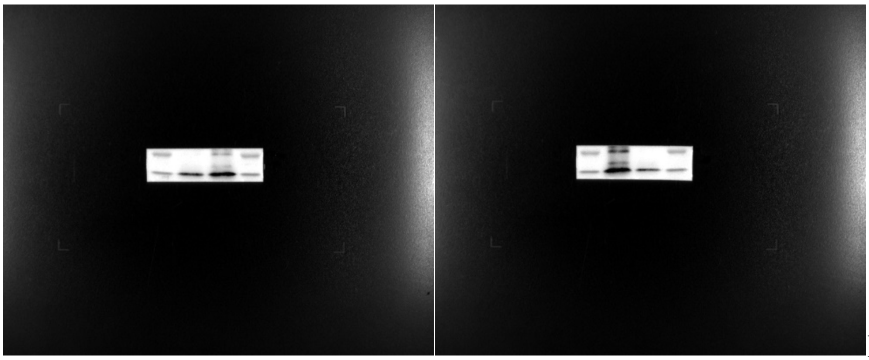

PHLDA2 17KD

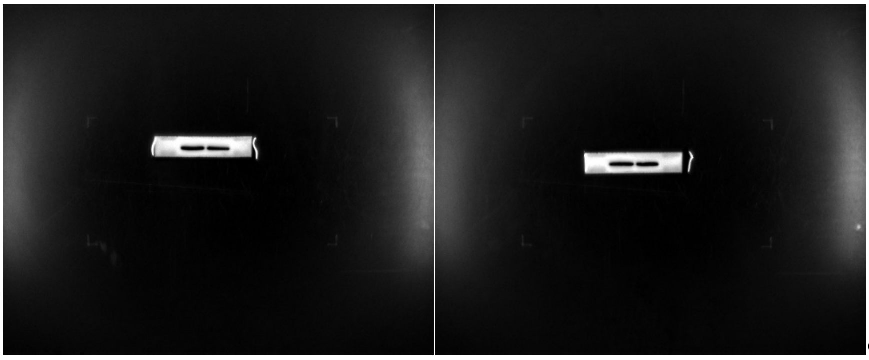

GAPDH 36KD

Figure 5I

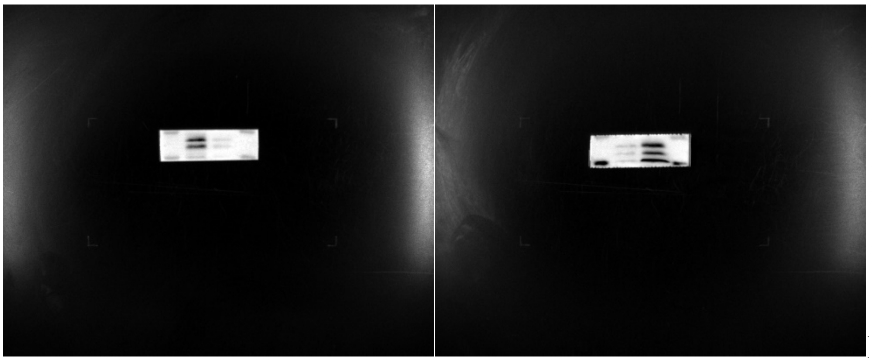

PHLDA2 17KD

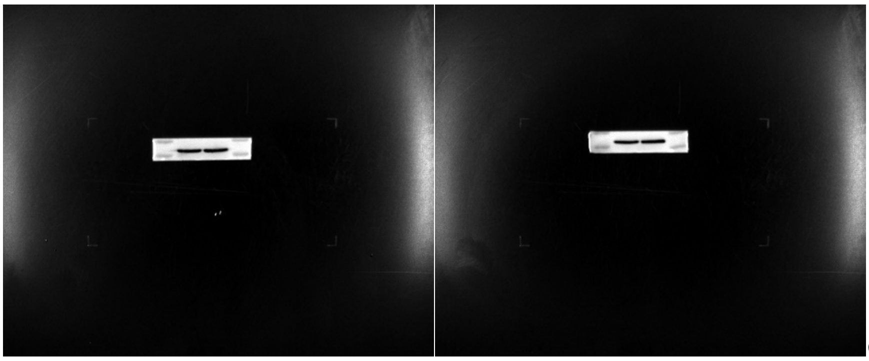

GAPDH 36KD

Figure 5J

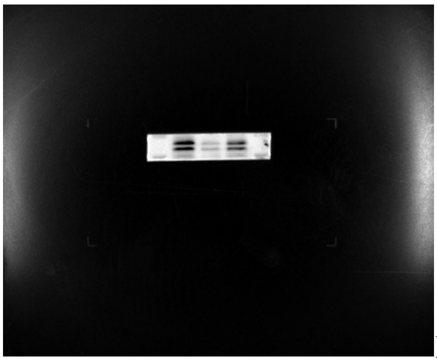

PHLDA2 17KD

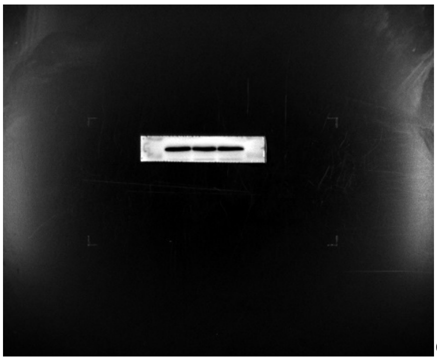

GAPDH 36KD

Figure 6F

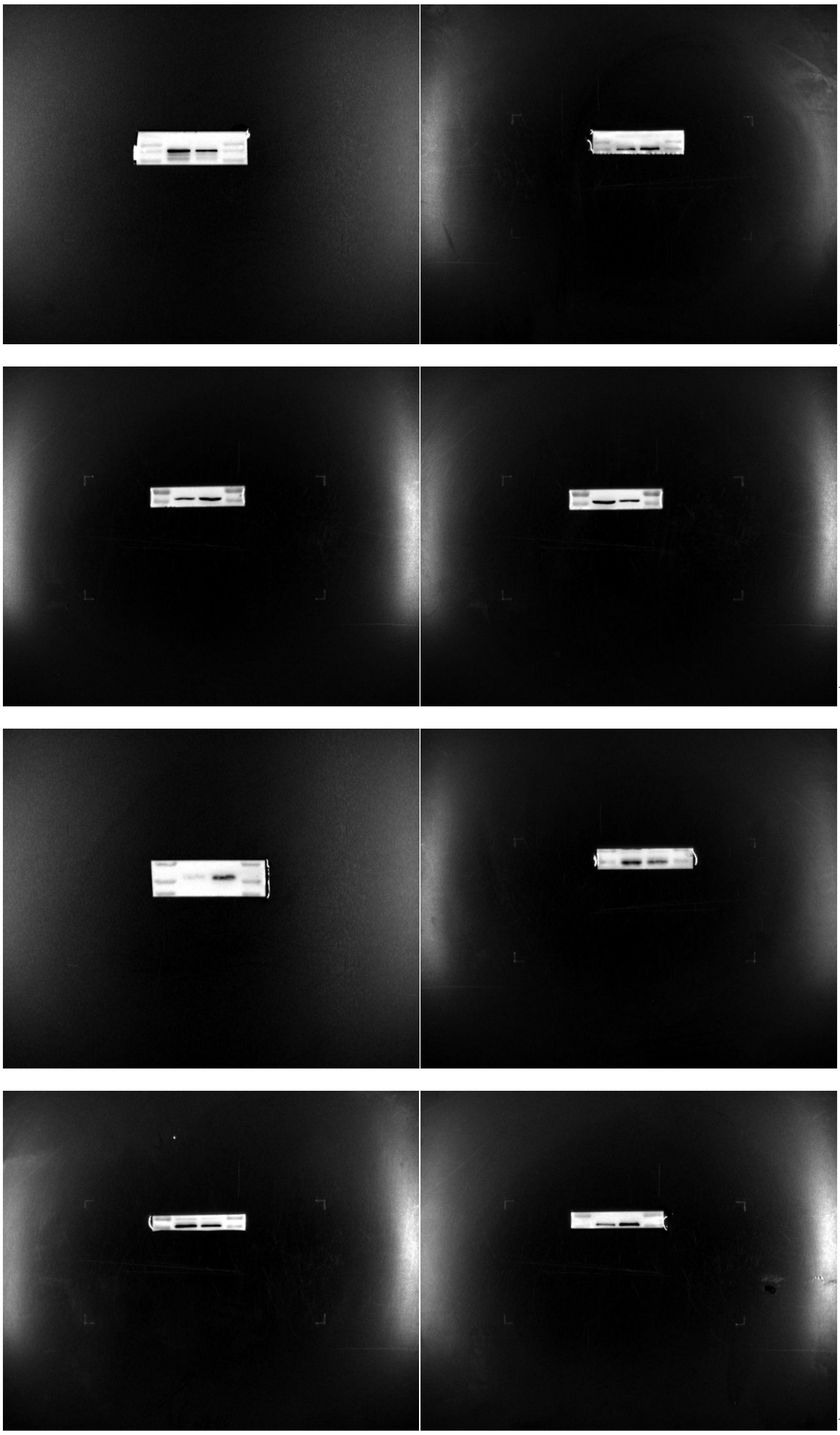

E-cadherin 135KD

Vimentin 56KD

Snail 30KD

p57 57KD

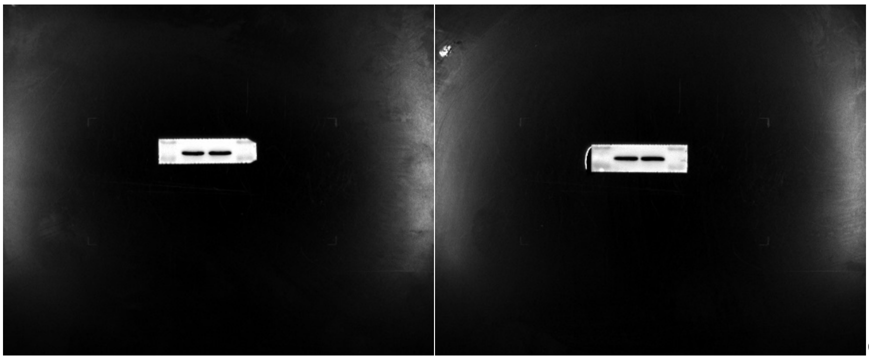
GAPDH 36KD

Figure 6G

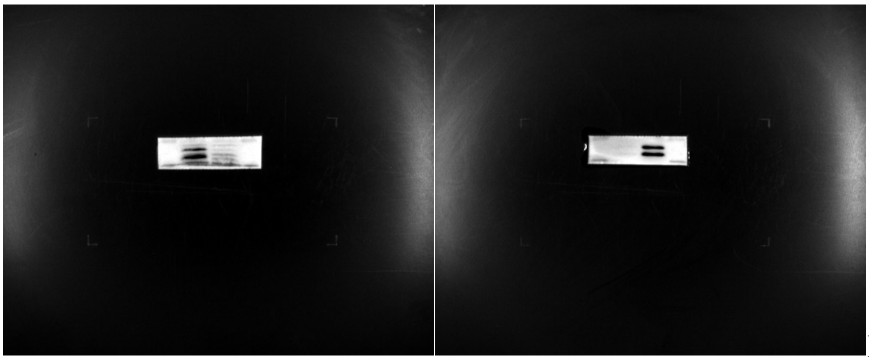
PHLDA2 17KD

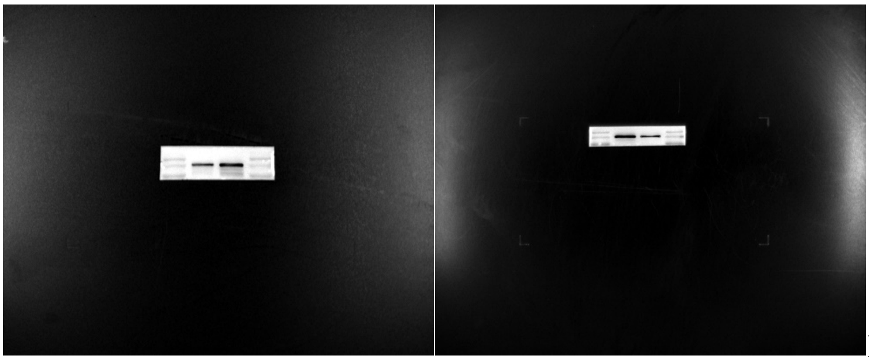
E-cadherin 140KD

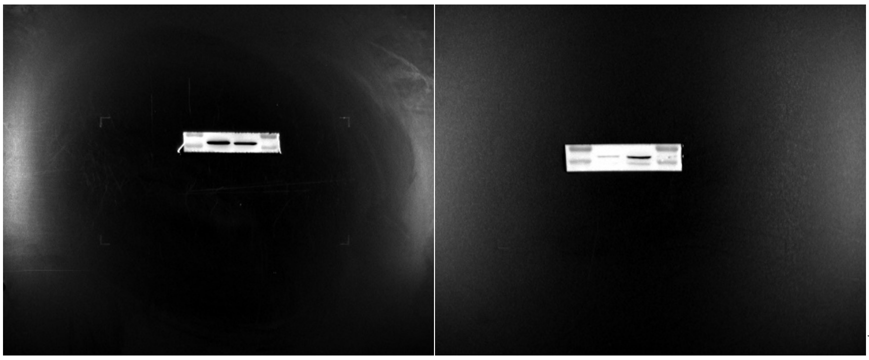
Vimentin 57KD

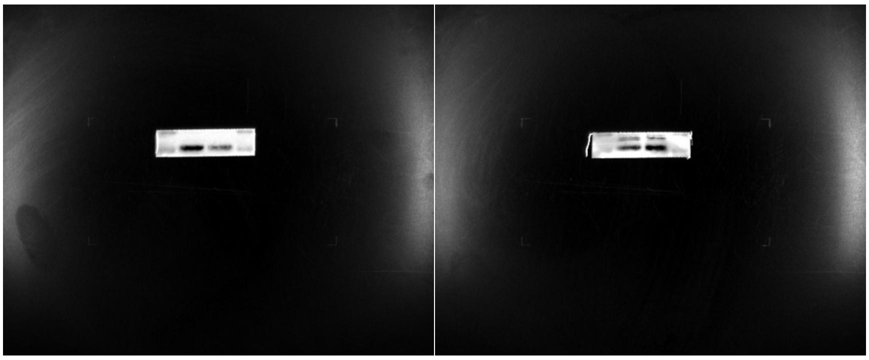
Snail 30KD

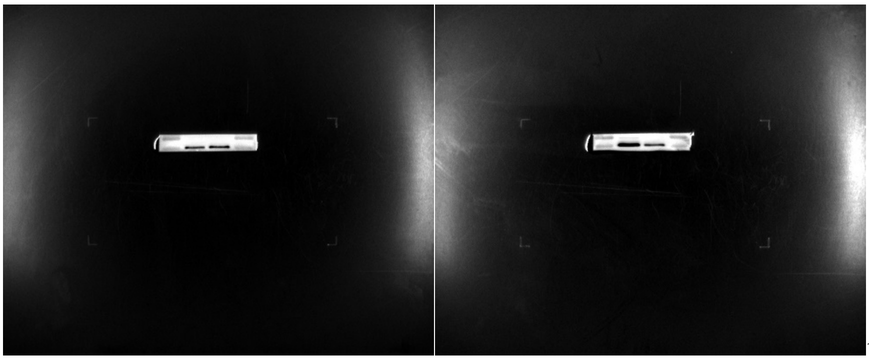
p57 57KD

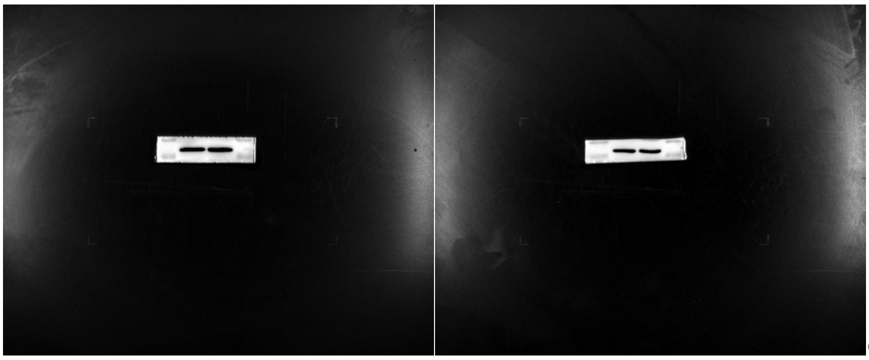
GAPDH 36KD

Figure 6H

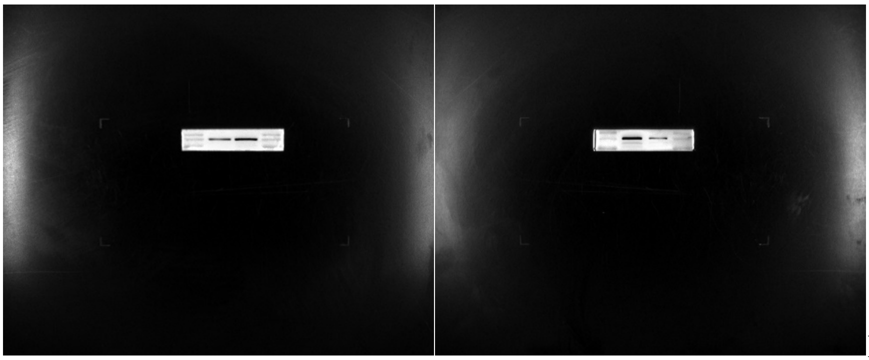

E-cadherin 135KD

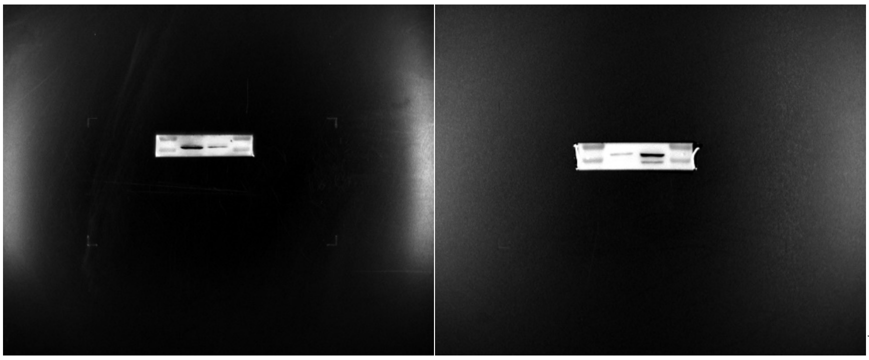

Vimentin 57KD

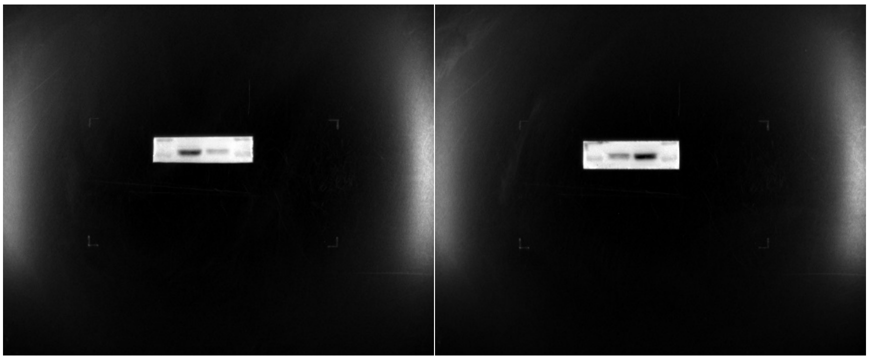

Snail 30KD

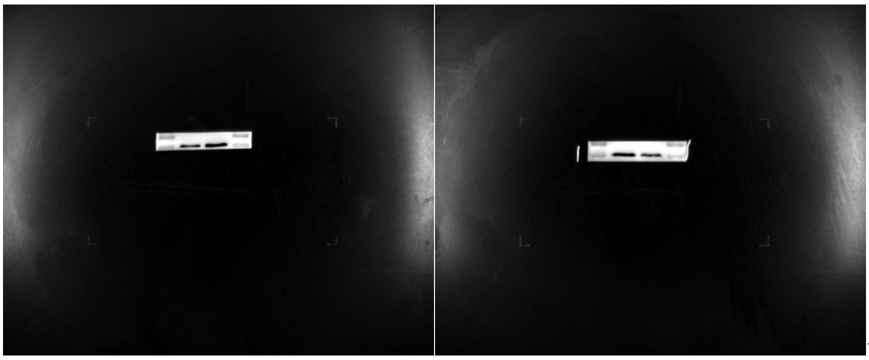

p57 57KD

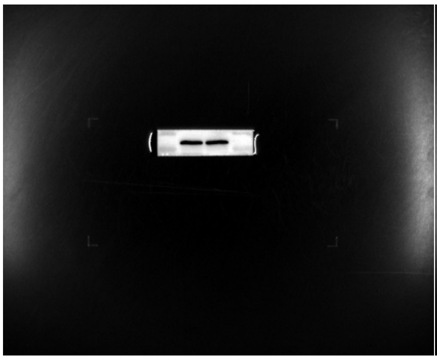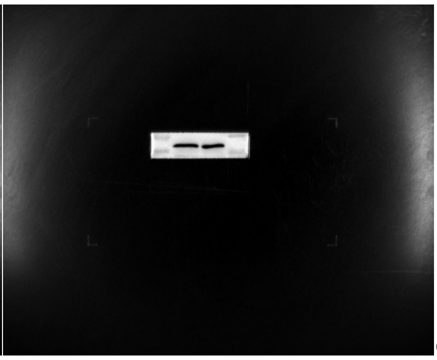

GAPDH 36KD

Figure 6I

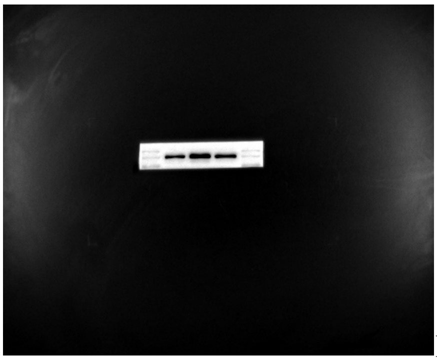

E-cadherin 135KD

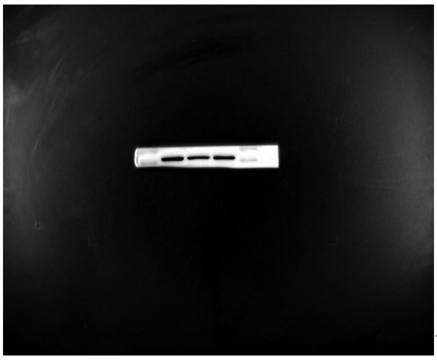

Vimentin 57KD

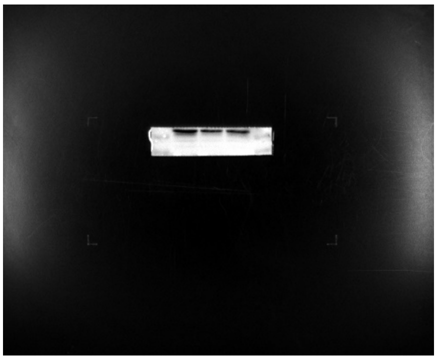

Snail 30KD

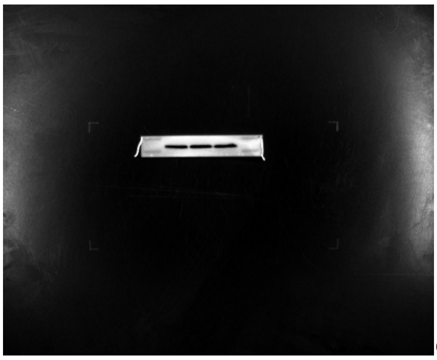
GAPDH 36KD
